# Electrocardiogram-Based Mental Stress Detection Amid Everyday Activities Using Machine Learning: Model Development and Validation Study

**DOI:** 10.2196/80450

**Published:** 2026-04-07

**Authors:** Buelent Uendes, Alex Antonides, Sjors van de Ven, Denise Johanna van der Mee, Eco de Geus, Mark Hoogendoorn

**Affiliations:** 1 Department of Computer Science, Vrije Universiteit Amsterdam, De Boelelaan 1111, Amsterdam, 1081 HV, The Netherlands, 49 15221457090; 2 Department of Biological Psychology, Vrije Universiteit Amsterdam, Amsterdam, The Netherlands

**Keywords:** mental stress, machine learning, electrocardiography, ECG, stress detection, generalizability

## Abstract

**Background:**

Frequent, sustained stress is linked to poor health and requires monitoring for early intervention. Electrocardiograms (ECG) are promising biomarkers because they can be recorded noninvasively and continuously using wearable devices. However, tracking stress with ECG is challenging because daily activities elicit responses similar to mental stress (MS), and various mental stimuli that individuals encounter complicate the use of machine learning (ML) models trained on a limited set of stressors.

**Objective:**

We (1) evaluated the ability of ML models to distinguish MS episodes from a composite “no-stress” background, including rest and low- to moderate-intensity activities; (2) assessed their generalizability to new stressors and participants; and (3) tested robustness to lower sampling rates and fewer features, to explore their suitability for lightweight wearables.

**Methods:**

We used a comprehensive ECG dataset sampled at 1000 hertz from 127 participants who underwent various mental stressors and engaged in diverse physical activities. A 30-second window was used to extract 55 features from time, frequency, nonlinear, and morphological domains. We trained a logistic regression (LR) model and an extreme gradient boosting (XGBoost) model, splitting the data into 60/20/20 for training, validation, and testing. Shapley additive explanation values were computed to explain model predictions. Additional analyses included leave-one-stressor-out; downsampling to 500, 250, and 125 hertz; a time-window sensitivity analysis; and reducing the number of features to as few as 5.

**Results:**

XGBoost achieved an area under the receiver operating characteristic curve (AUROC) of 0.741 (95% CI 0.701‐0.783) and an area under the precision-recall curve (AUPRC) of 0.706 (95% CI 0.658‐0.753), compared with 0.724 (95% CI 0.678‐0.772) and 0.691 (95% CI 0.639‐0.742) for LR. The mean performance difference between XGBoost and LR was 0.017 for AUROC (95% CI 0.001‐0.032) and 0.015 for AUPRC (95% CI −0.001 to 0.037; clustered bootstrap analysis using 2000 participant-level resamples), suggesting that LR performs comparably to the nonlinear XGBoost model. Both models were robust to downsampling and feature reduction (10 features retained >93% of performance). Extending the analysis window to 60 seconds improved model performance across all sampling rates, highlighting a trade-off between rapid detection and overall performance. When evaluating discrimination from physical activity, models achieved acceptable specificity for light physical activity (XGBoost: 0.787; LR: 0.794) but poor specificity for moderate physical activity (XGBoost: 0.418; LR: 0.444). Both models generalized to most unseen stressors, although performance varied across stressors, with limited transfer to the social-evaluative stressor. Feature importance analysis revealed fuzzy entropy and frequency-based features as key predictors.

**Conclusions:**

ML models can detect MS with high sensitivity and remain robust to lower sampling rates and fewer features. Generalization to novel stressors was stressor-dependent. Importantly, our results highlight challenges in distinguishing stress-related cardiac responses from those caused by physical exertion, revealing critical limitations of single-sensor ECG approaches for MS detection.

## Introduction

### Background

Mental stress (MS), an ever-present aspect of life, occurs when external demands exceed an individual’s available physiological and psychological coping resources [1,2]. While short-term exposure to stress may enhance focus and performance [3], frequent or prolonged exposure can adversely affect health, contributing to psychiatric conditions [4,5] and cardiovascular disease [6]. Consequently, accurate and timely detection followed by effective stress management is essential to mitigate these adverse health outcomes.

Traditional stress assessment methods, such as cortisol analysis [7], are burdensome. In contrast, self-report questionnaires, such as the Perceived Stress Scale [8], administered retrospectively or through ecological momentary assessment, offer greater convenience but are limited in temporal granularity [9]. Moreover, they may be susceptible to recall and reporting bias and less reliable among individuals with alexithymia. Effective stress detection, by contrast, should be minimally invasive and support continuous monitoring to capture early warning signs of stress [10].

Given that stress modulates the activity of the autonomic nervous system (ANS) [11], physiological biomarkers such as the electrocardiogram (ECG) and electrodermal activity have been widely proposed for automated stress monitoring. Furthermore, these signals are increasingly accessible through modern wearable devices, making real-time stress tracking more feasible. However, physiological signals often exhibit subtle and complex patterns that can be difficult to analyze and interpret using traditional statistical methods [1].

### Objectives

Machine learning (ML) has emerged as a powerful tool for analyzing high-dimensional data, excelling at uncovering patterns and complex relationships [12]. Consequently, ML has increasingly been used in recent studies to detect stress responses from the biomarkers mentioned above [13-16]. However, most existing studies exhibit one of the following limitations: (1) small cohorts; (2) a narrow range of mental stressors investigated; and (3) restrict MS detection to seated baseline (BL) conditions. This restriction leaves it unclear whether ML models can distinguish stress-induced ECG signals from those triggered by everyday movements, which often produce similar physiological responses. Moreover, because individuals encounter a wide range of novel stressors in daily life, models must generalize to stimuli absent from their training data. This aspect has been largely overlooked in prior work. The few studies that investigate generalization [17-19] typically train their ML models on one dataset and test them on another. Although informative, this approach conflates differences in participant demographics, sensor hardware, and stressor types, making it hard to isolate which factor drives any performance drop.

In this study, we address these gaps by developing and rigorously evaluating two ML models, namely logistic regression (LR) and extreme gradient boosting (XGBoost), on a large ECG dataset sampled at 1000 Hz. The dataset, collected from a controlled laboratory experiment at the Vrije Universiteit Amsterdam and published by van der Mee et al [20], included 127 participants who performed both diverse mental-stress tasks and everyday activities (eg, dishwashing and walking). In this study, we define “mental stress” as the physiological response elicited by cognitively or emotionally challenging tasks. Subsequently, we formulated a binary MS versus no-stress classification task, where the no-stress condition includes seated BLs, recovery periods, and low-to-moderate-intensity physical activities. This design not only improves ecological validity but also tests each model’s ability to distinguish stress‐induced ECG changes from those driven by physical exertion, without relying on additional sensors (eg, accelerometers). To assess generalization, we evaluate our models on unseen participants and on “novel” stressors, using a leave-one-stressor-out protocol, where we withhold each mental stressor during training and assess model performance on this specific stressor. We further investigate the robustness of our models to lower sampling frequency and the reduction of the feature set. Together, these experiments provide key insights for transitioning ECG-based MS detection from laboratory settings to real-world contexts.

## Methods

### Overview

The structure of the following section is in line with the TRIPOD+AI (Transparent Reporting of a Multivariable Prediction Model for Individual Prognosis or Diagnosis–Artificial Intelligence) statement [21,22]. Sections of the guideline not applicable to this research were omitted for brevity.

### Data

This study is a retrospective analysis of pseudonymized data collected from a controlled laboratory experiment conducted at the Vrije Universiteit Amsterdam and published by van der Mee et al [20]. The experimental conditions are shown in Table 1. In addition to ECG signals, subjective affect was assessed after each experimental condition using self-reported measures of positive and negative affect, as measured by the Maastricht Questionnaire [23]. We refer to the original study by van der Mee et al [20] for detailed explanations of the experimental procedure.

**Table 1.** Experimental conditions and their associated labels used in our study. The laboratory protocol consisted of mental stress (MS) tasks, physical activities at varying intensities, baseline (BL) rest, and recovery periods, each labeled for binary classification as MS or nonstress (BL, low physical activity [LPA], and moderate physical activity [MPA]). A total of 127 healthy adults participated in the experiments conducted in the Netherlands (data collected from 2017 to 2019).

Experimental condition	Duration (min)	Label
Standing	3	LPA
Sitting	3	BL
Tone avoidance	4	MS
Recovery (sitting)	2	BL
Short sing-a-song stress test (anticipatory)	1	MS
Recovery (sitting)	2	BL
Paced auditory serial addition test	4	MS
Recovery (sitting)	2	BL
Raven’s Progressive Matrices	4	MS
Walking at a natural pace	2	MPA
Recovery (standing)	2	LPA
Dishwashing	2	MPA
Vacuum cleaning	2	MPA
Recovery (sitting)	2	BL
Tone avoidance (repeat)	4	MS
Recovery (sitting)	2	BL
Paced auditory serial addition test (repeat)	4	MS
Recovery (sitting)	3	BL

### Participants

As outlined in the study by van der Mee et al [20], participants were required to be Dutch-speaking, employed or enrolled in school, and aged 18-48 years. Exclusion criteria included a BMI above 30, high cholesterol, diabetes, liver or thyroid disease, and use of medications that affect the ANS, such as antidepressants or anticholinergics. The final sample included 127 participants (71 female and 56 male) with a mean age of 23.3 (SD 5.6) years. We refer the reader to the original study for additional details on recruitment and population. The data used in this analysis were pseudonymized.

### Outcome

For this research, the different experimental conditions, as shown in Table 1, were categorized into the following labels:

- BL: This group includes conditions in which participants were primarily resting, such as sitting and recovery periods.
- MS: This group includes conditions aimed to induce cognitive or emotional stress, such as the tone avoidance (TA) reaction time task, the sing-a-song-stress test (SSST), the paced auditory serial addition task (PASAT), and the Raven’s Progressive Matrices (RAVEN) test. For the SSST, the labeled stress segments correspond to the anticipatory phase before singing, that is, the period during which participants are informed they will be asked to sing but have not yet begun.
- Low physical activity (LPA): Standing, including recovery periods that involve standing, is classified as LPA.
- Moderate physical activity (MPA): Activities involving moderate physical exertion, such as walking at a moderate pace, washing dishes, and vacuuming.

### Data Preparation

ECG signals are susceptible to various forms of noise and artifacts, which can obscure underlying cardiac activity and hinder accurate analysis. Thus, data preprocessing is crucial to ensure the quality and consistency of the input for the subsequent analysis.

In the first stage of data preprocessing, ECG segments unrelated to the laboratory conditions of interest were removed. These segments included the first and last minutes of the experimental setup, which captured the experimental setup and ECG lead removal, as well as any designated “short break” conditions. Compared with the original study, we excluded high-intensity physical activities (eg, treadmill running and stair climbing), as these activities are easily distinguishable from MS by, for instance, considering the maximum heart rate (HR) and could inflate reported model performance.

In the subsequent step, a 0.5 Hz high-pass Butterworth filter (order=5) was used to remove BL wander and slow drifts in the signal BL caused by respiration, movement, or electrode issues, as well as a power line filter (50 Hz) to attenuate noise from electrical power sources. Both filtering steps were implemented using the preprocessing pipeline provided by the NeuroKit2 package (version 0.2.7) [24]. While filtering addresses signal quality issues, some segments may remain unreliable due to factors such as temporary electrode detachment caused by leads accidentally falling off during the experiment. To identify and address these segments, a signal quality index (SQI) was computed for each QRS complex. Segments with an SQI below 25% were deemed unreliable and discarded. This threshold was chosen based on visual inspection, where segments with SQI values below this level often exhibited flat lines, rendering the identification of key peaks unreliable.

### Predictors

#### Overview

For each participant, the data was segmented using a sliding-window approach, and features were subsequently calculated. We used a 30-second window size with a step size of 10 seconds. This window length represents the minimum duration demonstrated in prior work to yield reliable heart rate variability (HRV) estimates [25], yet remains short enough for near-real-time applications. Furthermore, our window size aligns with previous research [26].

Following R-peak detection and waveform delineation of the ECG signal, various features were extracted from the resulting ECG signal based on prior research:

#### Time-Domain Features

For each time window, we extracted the normal-to-normal (NN) interval series, converted it to an instantaneous HR series (in beats per minute [BPM]), and then computed the mean, SD, minimum, and maximum of that series. These measures capture the central tendency and dispersion of the HR, which typically rise during physical exertion and in response to acute mental stressors [27].

HRV features are essential markers for ANS activity [25] and have been consequently linked to stress in prior research [28-30]. Therefore, we calculated the average value of the NN intervals (AVNN: average normal-to-normal), the SD of the NN intervals (SDNN), as well as the root-mean-square of successive differences (RMSSD) for each time window. We also included the ratio between the SDNN and the RMSSD, as it serves as a time-domain surrogate for the low-frequency to high-frequency power ratio, a standard marker of ANS activity [31,32].

In addition to features like AVNN and SDNN, we derived several complementary time‐domain features to capture both absolute and relative variability in NN intervals. These include NN20 and NN50, the counts of consecutive interval differences exceeding 20 milliseconds and 50 milliseconds, and their normalized counterparts, PNN20 and PNN50, as these features are helpful for stress monitoring [33,34].

To assess variability relative to mean HR, we calculated CVNN (SDNN/AVNN) and CVSD (RMSSD/AVNN). Finally, we quantified dispersion beyond SDs by computing the IQR of NN intervals and the median absolute difference of NN intervals.

#### Frequency-Domain Features

To assess the spectral characteristics of the ECG signal, Welch’s method was used to estimate the power spectral density across three frequency bands: high frequency (HF; 0.15‐0.40 Hz), very high frequency (VHF; 0.40‐0.50 Hz), and ultra-high frequency (UHF; 0.50‐1.00 Hz). Although prior studies commonly analyze only frequencies up to the HF band (0.40 Hz), this may be restrictive during stress, as acute psychological stress can elevate respiratory rate [35-37], potentially shifting the respiratory-driven modulation of R-to-R intervals above the HF band. Supporting this, Hernando et al [38] showed that respiratory rate increases and becomes less stable during stress, and that incorporating respiratory information improves the characterization of the stress-related autonomic response.

Motivated by this evidence, we included the VHF band (0.40‐0.50 Hz), as implemented in the NeuroKit2 toolbox [24], to capture high-frequency variability potentially associated with stress-related changes beyond the conventional HF range. We additionally investigated the UHF band (0.50‐1.00 Hz) in an exploratory manner, as this range may reflect high-frequency variability components that differ systematically across conditions. Lower-frequency bands, such as very low frequency (0.0033‐0.04 Hz), were not considered in this study, as their estimation would require longer time windows to be accurately captured [31].

Following the approach taken by Schmidt et al [13], we computed the absolute spectral power within each predefined frequency band. Then we expressed each band’s power as a fraction of the total spectral power to quantify its proportional contribution. We also extracted the minimum, maximum, mean, SD, and entropy for each frequency band in line with the approach taken by Karthikeyan et al [39].

#### Nonlinear Features

Given the inherent nonlinearity and complexity of the MS response [40], we included several nonlinear indices of HRV derived from NN intervals to capture the subtle fluctuations and irregularities in ANS activity. Following the approach taken in Tanev et al [41], we included the approximate entropy [42,43] and fuzzy entropy [44]. We excluded sample entropy [43] because approximate entropy is better suited to shorter time windows [45]. Furthermore, as a derivative of sample entropy, fuzzy entropy has been shown to outperform sample entropy [46].

Using Poincaré plot analysis, we extracted the SD1 and SD2 features, along with their ratio, to gain insights into the dynamics of short-term and long-term HRV [45]. Furthermore, heart rate asymmetry (HRA), that is, the asymmetrical distribution between accelerating and decelerating NN intervals, has been shown to reflect MS [47]. Consequently, we extracted the area index [48] as a feature of the HRA. In contrast to other HRA measures, such as Guszik’s slope or Porta’s index, the area index has been reported to exhibit fewer variations in short-term heartbeat intervals [48].

Because the recurrence quantification analysis has been shown to reflect the sympathetic and parasympathetic nervous system [49,50], we extracted three features from the recurrence quantification analysis plot: average length, longest length, and the entropy of the vertical line (*W*, *W*_max_, and *W*_En_).

To assess short-term correlations in the ECG signal, we used detrended fluctuation analysis and extracted the α_1_ value [51], which is moderated by sympathetic activity [52,53]. In contrast to the α_1_ value, which requires 3 to 16 beats for estimation, the α_2_ value needs 16 to 64 heartbeats [54], which may be problematic given our short 30-second analysis window and the variability in HR across experimental conditions. For this reason, we excluded the α_2_ from our feature set.

Last, we included the HR fragmentation features proposed by Costa et al [55], that is, percentage of short segments (PSS), inverse average length of segments (IALS), percentage of inflection points, and percentage of NN intervals in alternation segments, as these biomarkers have been proposed to capture the functionality of the HR control system.

#### Morphological Features

Beyond linear and nonlinear dynamics, morphological features (ie, characteristics that describe the shape, size, or timing of ECG waves) were included to capture further nuances in cardiac response during MS. In particular, we focused on T-wave alternans (TWA), a beat-to-beat variation in the amplitude of the T-wave that reflects subtle alterations in ventricular repolarization and which has been identified as a potential marker of MS [7,56].

**Figure 1.**
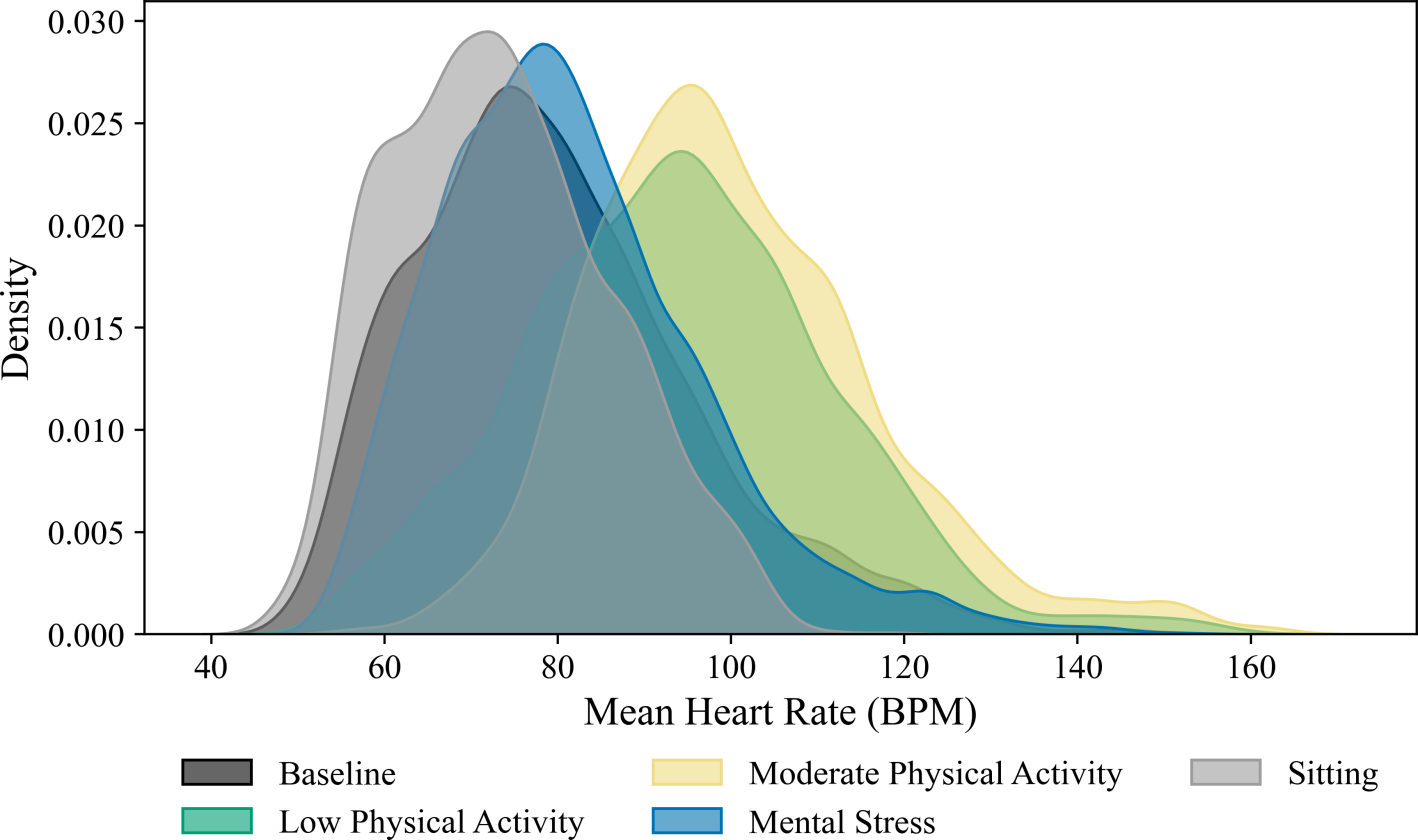
Heart rate distributions across experimental conditions (127 total participants). Kernel density plots show mean heart rate (BPM) during sitting, recovery (sitting), low physical activity, mental stress, and moderate physical activity. Overlapping distributions across experimental conditions highlight the difficulty of discriminating between mental stress and nonstress conditions using mean heart rate alone. BPM: beats per minute.

While the spectral method and the modified moving average (MMA) method are both widely used for TWA analysis, the latter was used to extract the TWA feature, as the MMA does not require participants to achieve a HR of 105 to 110 BPM [57]. This is crucial, as participants in the MS or seated BL conditions may experience HRs below this threshold (Figure 1).

Using the MMA approach [58], we delineated and extracted the T-wave from each 30-second ECG window, numbered them sequentially, and split them into odd (1, 3, …) and even (2, 4, …) beats. We then applied an MMA to each group and defined TWA as the maximum amplitude difference between the odd- and even-beat templates.

The complete set of features used in this study is presented in Multimedia Appendix 1 [55]. We excluded any 30-second window with fewer than 12 detected R peaks or with a mean HR outside the physiologically plausible range of 40‐220 BPM during feature calculation. This exclusion removed 0.9% of all time windows.

Models were trained using ECG-derived features only to test whether ECG alone is sufficient for stress detection and to enable deployment on any wearable device without requiring personal or demographic data.

### Sample Size

As illustrated in Table 2, the 30-second sliding-window approach with a 10-second shift yielded 31,408 observations.

**Table 2.** Distribution of electrocardiogram windows across experimental conditions for mental stress (MS) detection (31,408 thirty-second windows from 127 participants; 10-second sliding-window shift). Windows were labeled as MS or nonstress conditions: baseline (BL) rest, low physical activity (LPA), or moderate physical activity (MPA).

Experimental condition	Sample size	Overall percentage (%)
BL	10,140	32.28
LPA	3222	10.26
MPA	3703	11.79
MS	14,343	45.68
Total	31,408	100.00

### Missing Data

Due to the controlled, high-quality conditions of the laboratory-based ECG data collection and the preprocessing pipelines used in this study, missing values were scarce. Out of 31,408 analysis windows (Table 2), only 50 (0.16%) windows contained missing data, all of which were attributed to the TWA feature, which requires an adequate number of detected T-peaks for reliable computation. We imputed these missing values using a k-nearest neighbors approach with a k value of 5, where neighbors were selected using Euclidean distance across all 55 standardized (as described below) features, and the missing values were imputed using the mean values of the 5 nearest neighbors. Both the imputer and the scaler were fitted exclusively on the training set to prevent data leakage.

### Analytical Methods

#### ML Models

In this study, we trained an LR with L2 regularization and a nonlinear tree-based model, that is, an XGBoost [59] model. While the LR can only capture linear relationships, its inherent interpretability offers an advantage, especially in the health care domain, where interpretability is a key requirement [60]. By contrast, we selected a tree-based model as the nonlinear approach, given its widespread use in ECG-based stress and affect detection [13,61-63]. Specifically, we selected XGBoost because gradient-boosting methods have demonstrated strong performance across various tabular benchmarks, matching or surpassing neural network architectures [64,65]. By evaluating both ML models side-by-side, we can directly assess the trade-off between more interpretable linear models and more complex ones in the context of ECG-based MS detection. To confirm the stability of the results, we also included results from a random forest (RF) model to assess robustness.

#### Model Development

We partitioned participants into training (75/125 participants, 60%), validation (26/125 participants, 20%), and test (26/125 participants, 20%) cohorts. For continuous features, we used z-standardization, whereas for discrete features (NN20, NN50, and *W*_max_), we applied min-max normalization across the training dataset. We used global rather than participant-specific feature normalization, as global normalization enables assessment of whether ML models can generalize to unseen individuals without requiring per-user calibration data. Hyperparameters for the ML models (LR, XGBoost, and RF) were optimized using Bayesian optimization with Gaussian processes (Optuna version 4.2.1; Preferred Networks, Inc) over 25 iterations; the full hyperparameter tuning ranges and selected values are detailed in Multimedia Appendix 2.

#### Model Evaluation

We quantified model performance using the area under the receiver operating characteristic (AUROC) curve and the area under the precision-recall curve (AUPRC) curve—the former capturing overall class separation, while the latter emphasizes stress detection performance directly, which is of particular importance in our study. In line with [66], we interpret 0.60‐0.69 as moderate, 0.70‐0.79 as acceptable, 0.80‐0.89 as good, and ≥0.90 as excellent. To evaluate how the ML models would perform in practice, we also report the *F*_1_-score, as well as sensitivity and specificity. For the latter metrics, we used the threshold that maximized the *F*_1_-score on the validation dataset. We computed 95% CIs for all metrics using participant-level cluster bootstrapping (2000 bootstrap samples), in which each bootstrap sample resampled participants within the test dataset with replacement and included all corresponding observations for each selected participant.

#### Sampling-Rate Robustness

Our ECG recordings were acquired at 1000 Hz, which exceeds the rates of commonly used datasets [13,61,67]. Using the NeuroKit resampling method, we downsampled our ECG signal to 500, 250, and 125 Hz. This procedure quantifies the effects of reduced temporal resolution on model performance, facilitates direct comparison with earlier work, and evaluates the feasibility for consumer-grade devices, such as the Hexoskin Proshirt (Carré Technologies Inc) [68], which operates at lower sampling rates. Furthermore, investigating whether the performance of MS detection is affected by the sampling rate has important implications and considerations for the design of memory-efficient devices.

#### Time-Window Robustness

To investigate the trade-off between the desire to capture MS in near-real-time (ie, shorter time windows) and the requirement to have sufficiently long windows to generate reliable ECG features, we compared model performance using our standard 30-second segments against extended 60-second segments. Importantly, we used a 20-second time shift for the latter to maintain a constant overall window overlap across both settings. This setup allows us to subsequently investigate how the window size affects model performance.

#### Model Explainability and Calibration

To explain the predictions of our ML models, we computed Shapley Additive Explanations (SHAP) [69] values. SHAP values are based on a game-theoretic approach to calculate a feature’s importance concerning the overall model prediction. For the LR model, we report, in addition to the SHAP values, the model coefficients.

Model calibration (ie, how well the provided model probabilities align with the actual probabilities of the outcomes) is particularly critical in the health care domain [66,70,71]. We assess calibration using calibration curves, the Brier score [72], and the Brier skill score. To obtain the latter, we compare the Brier scores of our ML models to those of a simple majority classifier. These analyses enable us to evaluate not only the predictive performance but also the reliability of the probabilities provided by our ML models. Following [73], we consider a model with a Brier score <0.20 to be well-calibrated. We further use isotonic regression to post hoc calibrate the model probabilities.

#### Parsimony

As previously outlined, and motivated by prior research, we engineered a total of 55 features spanning time, frequency, nonlinear, and morphological domains to capture physiological responses to MS. However, manual feature generation is a time-consuming process. To quantify the trade-off between model simplicity and predictive performance, we evaluated model performance using progressively smaller feature sets. We applied forward selection to choose subsets of 5, 10, and 20 features by iteratively adding features that maximally improved the overall model performance, while retraining each model at each step of the feature selection process. Compared with alternative feature selection methods such as principal component analysis [74] or mutual information gain, which calculates dependency between the predictor and outcome variable, the forward-selection method directly optimizes for predictive performance during the feature selection process by iteratively adding features that provide the greatest incremental benefit. This process inherently accounts for feature dependencies, as each selection is made conditional on the features already included. Our progressive reduction from 55 to 5 features allows us to quantify the trade-off between model simplicity and performance, while evaluating the extent to which a compact feature subset can maintain classification performance. We note that the forward selection process was performed on a single validation split (see section “Model Development”). The specific feature subset composition may therefore vary across different validation splits. However, the relatively large sample size of our study (127 participants; 31,408 windows) may partially mitigate, though not fully eliminate, the risk of overfitting to a specific data split. Nevertheless, we caution against overinterpreting the feature subsets.

#### Generalizability Across Stressors

Although our dataset encompasses a variety of different mental stressors (social stressors, cognitive stressors, and active coping), individuals can face a vast majority of varying stressors in their daily lives. To assess whether our models learn a general MS pattern and can generalize to unseen stimuli, we used a leave-one-stressor-out scheme. For each stressor category, we trained models on all but the held-out mental stressor and tested their performance on the unseen stimulus. This analysis can thereby give valuable insights into our model’s ability to generalize to novel stress types, a critical requirement for deploying ML for MS detection to ambulatory settings.

We implemented the analysis in Python (version 3.11) and used artificial intelligence tools to accelerate the programming process. Importantly, all output was carefully reviewed, and the code was double-checked and tested for correctness. We release the source code used for this study publicly on GitHub [75].

### Class Imbalance

Although the class distribution between the MS and nonstress states (BL, LPA, and MPA) is relatively balanced (45.68% MS vs 54.32% nonstress; Table 2), the leave-one-stressor-out analyses exhibit more pronounced imbalance, as specific mental stressors are left out during training, resulting in a class distribution of approximately 33.33% MS versus 66.67% nonstress examples (when PASAT or TA stimuli are left out). To overcome the class imbalance, we applied the synthetic minority oversampling technique (SMOTE) [76] exclusively to the training set to upsample the minority class, that is, the MS condition. SMOTE is an oversampling method that creates new synthetic instances of the minority class by interpolating between neighboring minority examples. To maintain a consistent training pipeline, we also used SMOTE in the main analysis, although it had a negligible impact on model performance in the more balanced MS versus nonstress setting (AUROC with vs without SMOTE: 0.741 vs 0.742 for XGBoost; 0.724 vs 0.724 for LR).

### Model Output

We framed our primary outcome as a binary classification task: detecting MS episodes across all nonstress states, including seated BLs, recovery periods, and LPA to MPA. In this way, our nonstress class reflects both desk-bound environments, such as office work in front of a computer, as well as more mobile contexts, such as those found in the teaching or nursing profession. By incorporating both dynamic and seated non-MS conditions, our models must learn to distinguish ECG changes induced by MS from those resulting from resting or physical activity—an inherently more complex task than handling either condition alone.

### Ethical Considerations

Given that this study represents a retrospective analysis of precollected data, ethical approval was not required under the Dutch Medical Research Involving Human Subjects Act (WMO) [77]. However, the study from which the experimental data were obtained [20] was approved by the Medical Ethical Committee of the Vrije Universiteit Amsterdam Medical Center (METc VUmc #2017.374, ABR #NL62442.029.17). All participants provided written informed consent before the start of the experiment and were either compensated with research credits (for student participants) or with a €50 (US $57.92) gift voucher. All data were pseudonymized.

## Results

### Exploratory ECG Signal Analysis

Before evaluating our models, we first examine 2 fundamental ECG biomarkers, namely mean HR and AVNN reactivity, to illustrate the intrinsic challenge of detecting MS in everyday routine activities. Mean HR was selected for its simplicity of calculation from an ECG signal. In contrast, HR reactivity (or its inverse, AVNN reactivity) was chosen for its link to cardiac sympathetic and parasympathetic tone and its reliability in recognizing MS in experimental conditions [34]; however, because it relies on a controlled BL measurement, it cannot be defined in unconstrained, fully ambulatory settings.

Figure 1 shows the kernel density estimates of mean HR for five experimental conditions: seated BL, recovery (sitting), low- and moderate-intensity activity, MS, and high-intensity activity. As expected, increasing physical activity leads to a corresponding increase in mean HR. Crucially, the distribution for MS (blue) overlaps extensively with both low- and moderate-intensity activity (green and yellow) and even with the resting peaks (BL and recovery in gray). This pronounced overlap underscores the difficulty of disentangling MS-induced changes from those arising during everyday activity or recovery based on a simple measurement such as HR.

**Figure 2.**
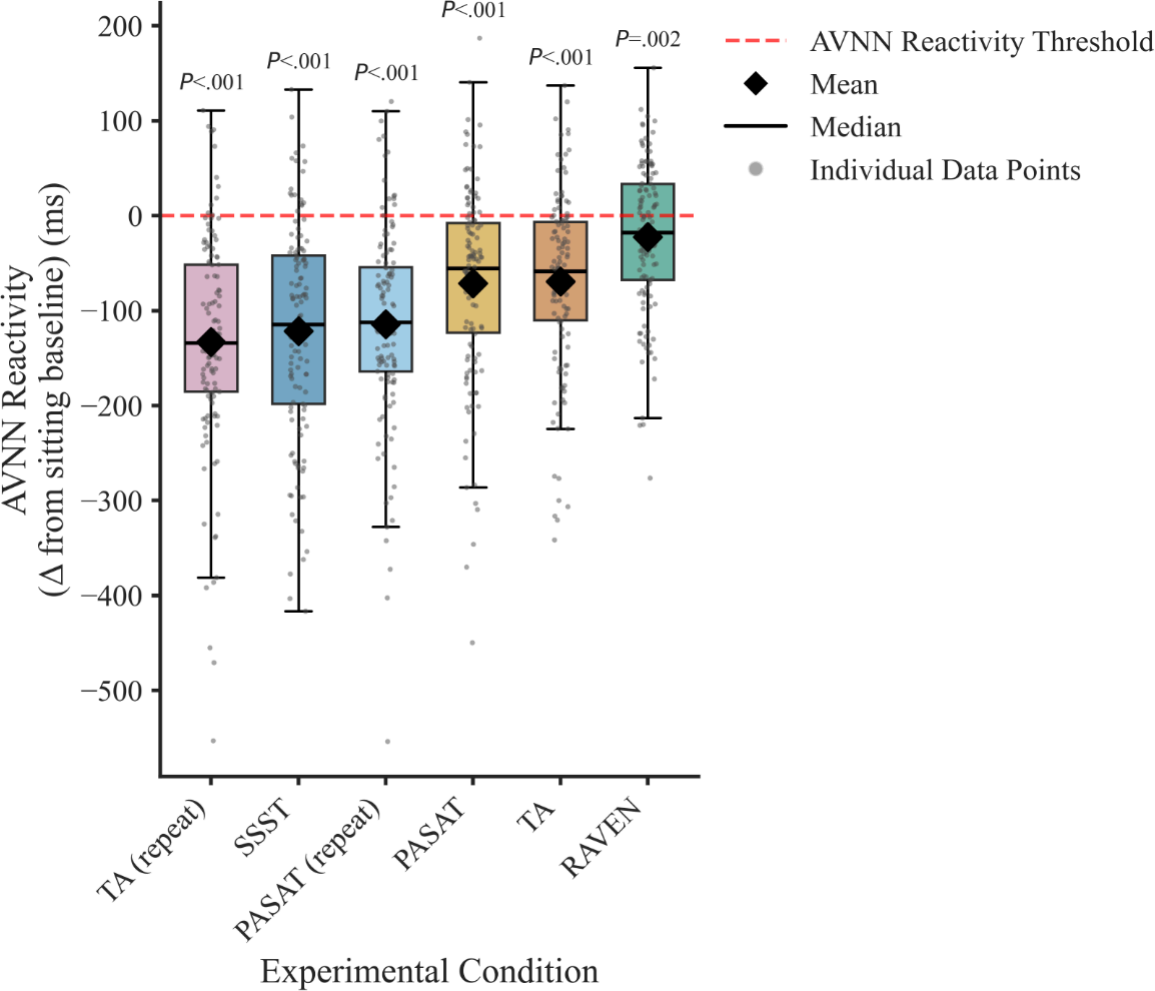
AVNN interval reactivity in ms across mental stressors. AVNN reactivity was calculated for each participant (127 participants) as the average NN interval during the mental stressor minus the average NN interval during the sitting baseline period (ie, the inverse of the commonly used HR reactivity). Statistical significance was assessed using a paired *t* test. Test statistics: PASAT: *t*_126_=–7.66, *P*<.001; PASAT (repeat): *t*_126_=–12.02, *P*<.001; RAVEN: *t*_126_=–3.19, *P*=.002; SSST: *t*_126_=–11.92, *P*<.001; TA: *t*_126_=–8.20, *P*<.001; TA (repeat): *t*_126_=–13.09, *P*<.001 for 2-sided paired *t* tests. AVNN: average normal-to-normal; HR: heart rate; ms: milliseconds; NN: normal-to-normal; PASAT: paced auditory serial addition task; RAVEN: Raven’s Progressive Matrices; SSST: sing-a-song-stress test; TA: tone avoidance.

Figure 2 shows the AVNN reactivity, calculated as the difference between AVNN during each experimental stressor and the seated BL, across all MS conditions. Although some individuals exhibit positive AVNN reactivity, represented by the mean change from the seated BL, was significantly negative across all tasks, indicating an overall decline in AVNN compared with BL (PASAT: *t*_126_=−7.66, *P*<.001; PASAT [repeat]: *t*_126_=−12.02, *P*<.001; RAVEN: *t*_126_=−3.19, *P*=.002; SSST: *t*_126_=−11.92, *P*<.001; TA: *t*_126_=−8.20, *P*<.001; TA [repeat]: *t*_126_=−13.09, *P*<.001 for 2-sided paired *t* tests). However, the magnitude of this change varied considerably across experimental conditions. For instance, TA repeat elicits the most considerable mean AVNN reduction, whereas RAVEN matrices produce a more modest decline.

Taken together, these figures demonstrate that neither mean HR nor HR reactivity (or its inverse AVNN reactivity) alone can reliably discriminate MS from everyday activity or account for heterogeneity across stress tasks and individuals. This motivates our extraction of 55 time-, frequency-, nonlinear, and morphological ECG features, and the use of ML models to develop an MS classifier that detects physiological patterns characteristic of MS.

The large interindividual variability in ECG responses underscores that MS reactions are person-specific, likely influenced by subjective perceptions of stress. In Multimedia Appendix 3, we also show changes in both positive and negative affect relative to the sitting BL. As expected, positive affect significantly decreased across all mental stressors (PASAT: *t*_126_=−13.25, *P*<.001; PASAT (repeat): *t*_126_=−13.45, *P*<.001; RAVEN: *t*_126_=−7.96, *P*<.001; SSST: *t*_126_=−4.65, *P*<.001; TA: *t*_126_=−7.71, *P*<.001; TA [repeat]: *t*_126_=−5.00, *P*<.001 for 2-sided paired *t* tests). Likewise, negative affect significantly increased compared with the sitting BL (PASAT: *t*_126_=−12.00, *P*<.001; PASAT [repeat]: *t*_126_=−6.57, *P*<.001; RAVEN: *t*_126_=−6.09, *P*<.001; SSST: *t*_126_=−6.67, *P*<.001; TA: *t*_126_=−8.31, *P*<.001; TA [repeat]: *t*_126_=−3.21, *P*=.002 for 2-sided paired *t* tests). Nonetheless, apparent individual differences emerged, highlighting the challenge of building models that generalize across both stressors and individuals.

### Classification Performance

#### Model Performance Across Sampling Rates

**Figure 3.**
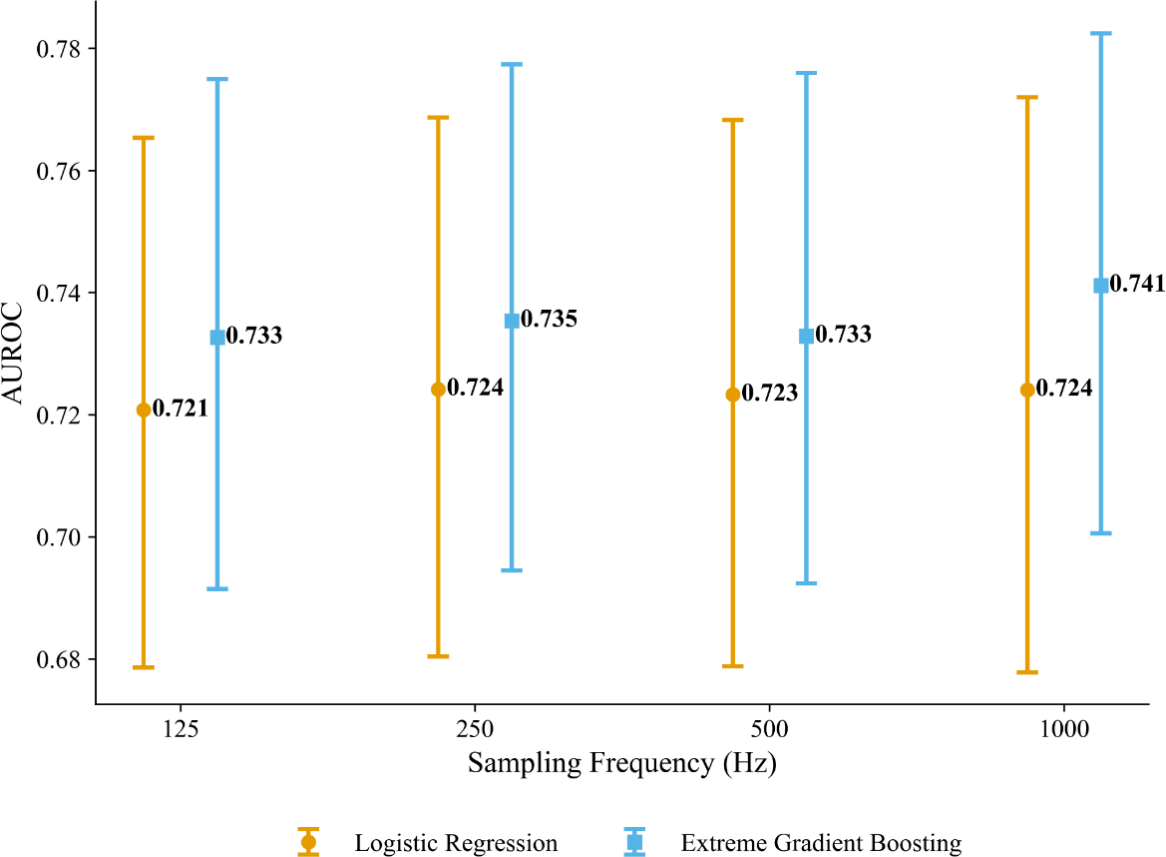
Performance comparison of logistic regression and extreme gradient boosting for electrocardiogram-based mental stress classification across sampling rates (127 total participants, 26 test set participants). Points represent bootstrapped mean AUROC with 95% CIs (error bars) from 2000 participant-level bootstrap samples. Models were trained on 55 features extracted from 30-second windows (10-second shift) using 60/20/20 (train/validation/test) splits at the individual level. Both models demonstrate robustness to downsampling from 1000 to 125 Hz. AUROC: area under the receiver operating characteristic curve.

Figure 3 compares LR and XGBoost on our MS detection task at 1000 Hz and after downsampling to 500, 250, and 125 Hz. Both classifiers achieve acceptable AUROC scores, with XGBoost outperforming the LR counterpart across all frequencies (XGBoost: 0.741, 95% CI 0.701‐0.783; LR: 0.724, 95% CI 0.678‐0.772) at 1000 Hz. Interestingly, AUROC performance remains stable across all sampling rates for both models, suggesting that sampling at higher rates does not yield performance gains. In Multimedia Appendix 4, we report corresponding AUPRC scores, which show similar trends and qualitative results. To estimate the performance difference between XGBoost and LR, we computed the mean pairwise differences in AUROC and AUPRC using 2000 participant-level cluster bootstrap samples, alongside 95% and 99% CIs to quantify uncertainty around these estimates. The results are reported in Multimedia Appendix 5. We chose the bootstrap approach over the DeLong test [78] because the latter assumes independent samples [79], which is violated in our setup due to within-participant correlation among the time windows. As shown in Multimedia Appendix 5, the mean performance difference (*Δ*) between XGBoost and LR was 0.017 for AUROC (95% CI 0.001‐0.032) and 0.015 for AUPRC (95% CI −0.007 to 0.037), using 2000 bootstrap samples. These differences are small in magnitude (<0.02), suggesting that both models perform comparably across sampling rates. At the *F*_1_-maximizing threshold (1000 Hz), XGBoost achieved a sensitivity of 0.800 (95% CI 0.698‐0.889) but a specificity of only 0.512 (95% CI 0.420‐0.609); LR performed similarly (sensitivity: 0.782, 95% CI 0.671‐0.878; specificity: 0.509, 95% CI 0.417‐0.613). This asymmetry indicates that, although both models reliably detect MS, approximately half of the nonstress segments are misclassified, highlighting a critical challenge for deployment. Complete results across all sampling rates, along with *F*_1_-scores and RF results, are provided in Multimedia Appendix 6.

#### Model Performance Under Simplified Conditions

To assess the impact of our inherently more challenging classification setup, we retrained our ML models on a simplified task: distinguishing MS from a seated BL. The corresponding results are reported in Multimedia Appendix 7. As can be seen, model performances improve substantially under these simplified settings: bootstrapped mean AUROC for XGBoost increases from 0.741 to 0.766, and from 0.724 to 0.752 for the LR at a sampling rate of 1000 Hz (with similar increases for the other sampling rates). The corresponding AUPRC improvements are even more substantial, underlining the challenge our setup poses for MS detection (especially without reliance on accelerometers).

As physiological recovery from stressors is not instantaneous, the recovery periods following each experimental condition (Table 1) likely contain residual autonomic arousal. To investigate the impact of this on model performance, we conducted a sensitivity analysis excluding recovery periods from the nonstress class. The AUROC performance remained stable (LR: 0.728, 95% CI 0.671‐0.787; XGBoost: 0.751, 95% CI 0.699‐0.806 at sampling rate of 1000 Hz), whereas the AUPRC improved substantially for both models (LR: 0.825, 95% CI 0.782‐0.867; XGBoost: 0.848, 95% CI 0.811‐0.885), suggesting that including recovery periods introduced label noise negatively affecting AUPRC, likely due to residual autonomic arousal from preceding stressors. However, because distinguishing stress from poststress recovery is an important and realistic challenge in detecting MS in everyday life, we retained recovery periods in the main analysis.

#### Time-Window Robustness

In Multimedia Appendix 8, we compare AUROC and AUPRC scores for 30-second versus 60-second windows for both models. Extending the time window yields consistent performance gains for both LR and XGBoost across all frequencies, highlighting the trade-off between quick stress detection (short time windows) and improved model performance (longer windows). For instance, at a 1000 Hz sampling rate, doubling the window from 30 to 60 seconds increased XGBoost’s bootstrapped mean AUROC from 0.741 to 0.759 (and AUPRC from 0.706 to 0.742), while LR’s mean AUROC rose from 0.724 to 0.747 (AUPRC from 0.691 to 0.730).

#### Instance-Level Agreement

We further assessed Cohen kappa regarding the model agreement on individual test instances. The substantial agreement of 0.735 (95% CI 0.695‐0.775) suggests that LR and XGBoost largely agree on an instance-level, which ECG responses reflect MS.

### Model Explainability and Model Calibration

**Figure 4.**
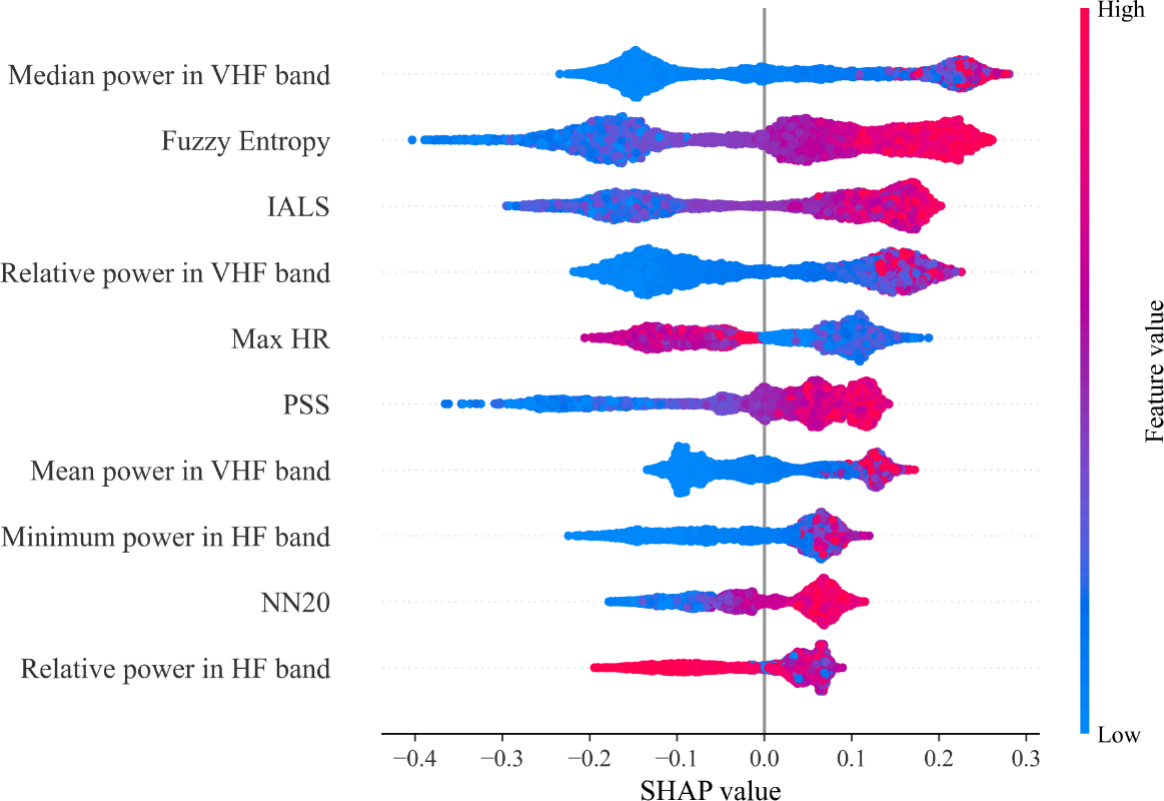
SHAP plot for the detection of mental stress versus no-mental-stress for the extreme gradient boosting model. The top 10 most important features are presented in descending order of importance. Each dot represents a Shapley value for a specific instance and feature, with the color indicating the underlying feature value ranging from high (red) to low (blue). HF: high frequency (0.15-0.40 Hz); HR: heart rate; IALS: inverse average length of segments; NN20: number of (normal-to-normal) NN intervals differing by more than 20 milliseconds; PSS: percentage of short segments; SHAP: Shapley additive explanation; VHF: very high frequency (0.40-0.50 Hz).

We next examined feature importance and model calibration, that is, how well the predicted probabilities represent the true probability of the outcomes. Figure 4 shows the 10 most important predictors for mental-stress detection in the XGBoost model according to the SHAP values. Notably, 5 out of these features originate from the frequency domain, highlighting the importance of spectral features in stress detection. Two heart-rate fragmentation indices (PSS and IALS) and fuzzy entropy also rank highly, with higher values of these nonlinear features pushing XGBoost’s output towards the MS class.

Interestingly, maximum HR and NN20, 2 simple time-domain features, are also important for predicting MS, demonstrating the relevance of time-domain metrics.

In Multimedia Appendix 9, we present the corresponding SHAP values for the LR model, along with its feature rankings based on the absolute values of the model coefficients. Fuzzy entropy emerges as the most important predictor of MS, underscoring its significance.

IALS again appears to be a crucial feature for MS detection, thereby confirming the importance of heart-rate fragmentation indices and underscoring the results obtained with the XGBoost model. Among time and frequency domain features, total and maximum power in the HF band, SD of HR, and PNN20 are also of high importance for the LR model.

**Figure 5.**
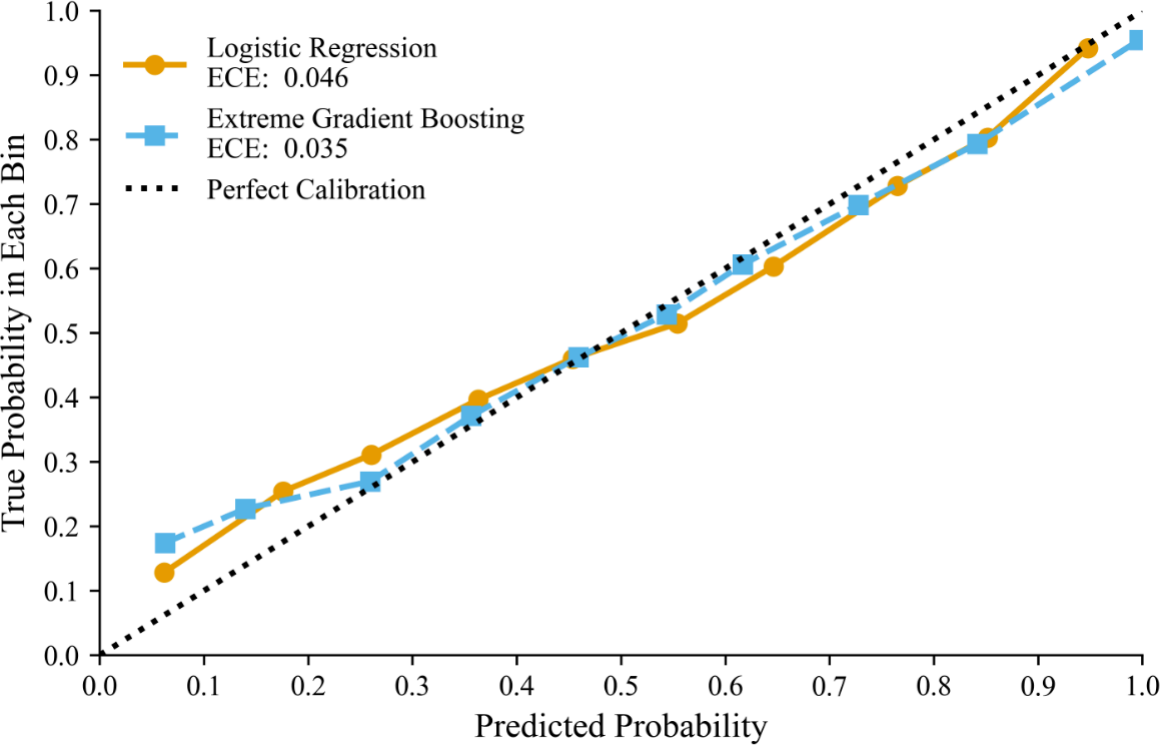
Calibration curves for the logistic regression and extreme gradient boosting models, evaluated on the held-out test set (127 total participants and 26 test participants). Points show the predicted model probabilities for mental stress versus the observed probability of mental stress (y-axis), across 10 bins. The dotted line indicates perfect calibration. ECE quantifies deviation from perfect calibration, with lower values indicating better calibration. ECE: expected calibration error.

The corresponding model-explainability results for the MS versus the seated nonstress condition (Multimedia Appendix 9; Figures 3-5) also highlight the importance of fuzzy entropy, as well as both the high-frequency and very high-frequency components, as predictive features.

In the health care domain, accurate probability estimates are crucial for informed decision-making [66,70]. For this reason, we assessed model calibration using the Brier score and the expected calibration error with 10 bins.

LR achieves a bootstrapped mean Brier score of 0.215 (95% CI 0.199‐0.232), while XGBoost scored 0.209 (95% CI 0.194‐0.224), thereby falling just above our <0.20 cutoff for well-calibrated probabilities. However, the AUROC for the XGBoost model is 0.615 (95% CI 0.586‐0.643), and 0.603 (95% CI 0.572‐0.633) for the LR, indicating substantial relative improvements over the dummy classifier BL that predicts the majority class (MS). Figure 5 shows the calibration curves for both classifiers, indicating slight underconfidence at low prediction probabilities (0.05‐0.25).

### Feature Parsimony

**Figure 6.**
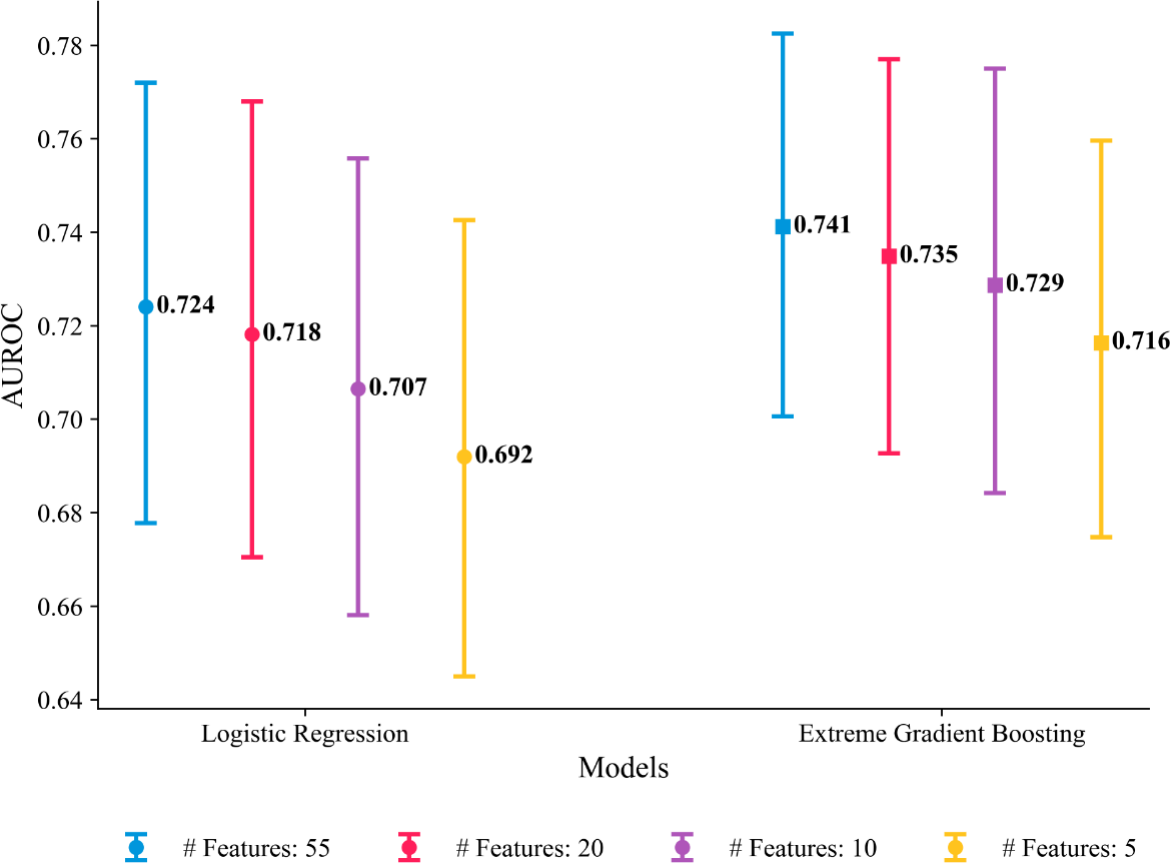
Performance comparison of logistic regression and extreme gradient boosting for electrocardiogram-based mental stress classification across varying feature-set sizes on the held-out test set (127 total participants and 26 test participants). Points represent bootstrapped mean AUROC with 95% CIs (error bars) from 2000 participant-level bootstrap samples. Models were trained using a 60/20/20 (train/validation/test) split at the individual level, with features selected via forward selection based on validation performance. Both models demonstrate robustness to feature reduction, maintaining >95% of original performance with only 10 features (logistic regression: 0.707; extreme gradient boosting: 0.729) compared with the full 55-feature set (logistic regression: 0.724; extreme gradient boosting: 0.741). AUROC: area under the receiver operating characteristic curve.

Generating the full set of 55 features is time-consuming and increases memory demands, which are both undesirable for resource-constrained wearable devices. Moreover, a large feature set can obscure model interpretability. To assess the trade-off between model simplicity and predictive performance, Figure 6 reports the AUROC as the number of features is progressively reduced. Notably, performance remains surprisingly robust, with over 95% of the original AUROC retained for both models, even with only 10 features. Interestingly, XGBoost outperforms the LR model across all feature sets. In Multimedia Appendix 10, we report the corresponding results for the AUPRC score, which are in line with the previously stated insights. The full list of selected features for each feature set is provided in Multimedia Appendix 11. As noted in the Methods section, feature selection was conducted using a single validation split. The resulting feature rankings should therefore be interpreted with caution.

### Generalization to Unknown Stressors

Every day, life exposes individuals to a wide variety of mental stressors, making it practically infeasible to train a model on every possible type of stimulus. Thus, a model’s ability to perform well under unseen mental stressors is essential for real-world deployment [17]. Table 3 reports the AUROC for each model under a leave-one-stressor-out scheme. For each held-out stressor, the model is trained on all remaining stressors plus the nonstress condition and then tested on its ability to discriminate the held-out stressor from the nonstress condition. Note that if a mental stressor had a repeat condition in the experimental protocol (eg, TA and PASAT), both stressors were left out during training of the ML models. To ensure these results accurately reflect the difficulty of generalizing to an unseen stressor, rather than just the effect of having fewer stressor types and training examples, we also report, for each left-out mental stressor, the BL AUROC on the remaining (known) stressors.

**Table 3.** Bootstrapped mean area under the receiver operating characteristic curve (AUROC) with 95% CIs from 2000 participant-level bootstrap samples for binary classification of mental stress versus nonstress conditions under leave-one-stressor-out evaluation on the held-out test set (127 total participants and 26 test participants). Models were trained using a 60/20/20 (train/validation/test) split at the participant level, using a total of 55 features. For each row, the “baseline” column reports the AUROC performance of the model on stressors included in the training set. In contrast, “left-out” reports the AUROC model performance on the held-out (unseen) mental stressor, assessing each model’s ability to generalize to novel mental stress conditions. Note that if a mental stressor had a repeat condition in the experimental protocol (eg, tone avoidance and paced auditory serial addition task), both stressors were left out during training of the machine learning models, which is why their baseline scores are identical.

Left-out mental stressor	AUROC model performance, 95% CI					
	LR^a^ baseline	LR left-out	XGBoost^b^ baseline	XGBoost left-out	RF^c^ baseline	RF left-out
SSST^d^	0.7218 (0.6812‐0.7660)	0.5620 (0.4667‐0.6548)	0.7360 (0.6975‐0.7759)	0.5600 (0.4795‐0.6380)	0.7413 (0.7039‐0.7792)	0.5499 (0.4680‐0.6261)
RAVEN^e^	0.7146 (0.6789‐0.7520)	0.6365 (0.5736‐0.6976)	0.7365 (0.7070‐0.7671)	0.6595 (0.6057‐0.7114)	0.7371 (0.7061‐0.7696)	0.6645 (0.6092‐0.7173)
PASAT^f^	0.7219 (0.6777‐0.7659)	0.7035 (0.6390‐0.7673)	0.7322 (0.6888‐0.7752)	0.7125 (0.6490‐0.7807)	0.7333 (0.6906‐0.7754)	0.7150 (0.6520‐0.7816)
PASAT (repeat)	0.7219 (0.6777‐0.7659)	0.6570 (0.5711‐0.7456)	0.7322 (0.6888‐0.7752)	0.6691 (0.5897‐0.7518)	0.7333 (0.6906‐0.7754)	0.6782 (0.5985‐0.7593)
TA^g^	0.7123 (0.6705‐0.7558)	0.7731 (0.7070‐0.8352)	0.7107 (0.6710‐0.7528)	0.7594 (0.6965‐0.8192)	0.7087 (0.6713‐0.7492)	0.7608 (0.7017‐0.8205)
TA (repeat)	0.7123 (0.6705‐0.7558)	0.7337 (0.6488‐0.8127)	0.7107 (0.6710‐0.7528)	0.7200 (0.6454‐0.7936)	0.7087 (0.6713‐0.7492)	0.7618 (0.6834‐0.8370)

a LR: logistic regression.

b XGBoost: extreme gradient boosting.

c RF: random forest.

d SSST: sing-a-song-stress test.

e RAVEN: Raven’s Progressive Matrices.

f PASAT: paced auditory serial addition task.

g TA: tone avoidance.

As shown in Table 3, model performance on held-out stressors generally declines compared with stressors included in the training set, as expected. Nevertheless, all models achieve AUROC well above the 0.50 chance level on most unseen stressors, confirming their ability to generalize to new mental stimuli. The exception was SSST, for which the 95% CI overlaps chance performance. Notably, the magnitude of performance drop to unseen mental stressors varies across the different stimuli. While both the SSST and RAVEN are most difficult to classify correctly as MS, the opposite holds true for the TA condition. The fact that LR, XGBoost, and the RF model show comparable generalization capability suggests that differences in generalizability to novel stressors arise from the underlying ECG response, rather than from model complexity.

Multimedia Appendix 12 presents AUPRC results that closely mirror the AUROC findings. SSST and RAVEN are the hardest to detect when excluded from model training. Although model performance is generally worse for the left-out stressor, the TA stressor (and its repeat condition) shows the opposite pattern: the AUPRC score when TA is held out exceeds that of other mental stressors included in training. This suggests that TA produces particularly distinctive and readily identifiable stress patterns.

### Stratified Stressor Analysis

While our leave-one-out evaluation results suggest that our ML models can overall generalize to novel mental stressors, the generalizability to unseen mental stressors varied significantly. In particular, the sharp contrast between TA and SSST, 2 mental stressors that elicit strong physiological responses as measured by AVNN reactivity (Figure 2), is intriguing. We therefore examined how each model performed on individual stressor types. Table 4, therefore, reports AUROC scores stratified by mental-stressor condition. Again, model performance varies by mental stressor: TA achieves AUROC>0.75, while others (eg, RAVEN and SSST) yield only moderate performance, which suggests that certain MS stimuli produce more distinguishable ECG responses (as compared with the nonstress class) than others. Importantly, this observation holds for LR, XGBoost, and RF alike, demonstrating that these differences are not related to model complexity. The results for the AUPRC, as presented in Multimedia Appendix 13, align with these findings.

**Table 4.** Bootstrapped mean area under the receiver operating characteristic curve (AUROC) with 95% CIs from 2000 participant-level bootstrap samples for mental stress classification, stratified by individual stressor type and evaluated on the held-out test set (26 participants from 127 total participants). Results are shown for logistic regression (LR), extreme gradient boosting (XGBoost), and random forest (RF) trained using 60/20/20 (train/validation/test) splits at the participant level with 55 features. Each row presents model performance for a specific mental stressor, assessing how well the models detect that stressor. An AUROC score of 0.50 represents chance-level performance (ie, a random baseline that predicts the majority class).

Mental stressor	AUROC model performance, 95% CI		
	LR	XGBoost	RF
SSST^a^	0.5902 (0.4955‐0.6792)	0.5726 (0.4958‐0.6464)	0.5690 (0.4926‐0.6421)
RAVEN^b^	0.6657 (0.6090‐0.7230)	0.6829 (0.6350‐0.7312)	0.6893 (0.6429‐0.7348)
PASAT^c^	0.7087 (0.6435‐0.7745)	0.7231 (0.6607‐0.7882)	0.7219 (0.6588‐0.7871)
PASAT (repeat)	0.6667 (0.5809‐0.7558)	0.6846 (0.6063‐0.7637)	0.6900 (0.6099‐0.7871)
TA^d^	0.7998 (0.7461‐0.8540)	0.8176 (0.7729‐0.8603)	0.8164 (0.7692‐0.8618)
TA (repeat)	0.7737 (0.6960‐0.8446)	0.7984 (0.7290‐0.8614)	0.7962 (0.7262‐0.8635)

a SSST: sing-a-song-stress test.

b RAVEN: Raven’s Progressive Matrices.

c PASAT: paced auditory serial addition task.

d TA: tone avoidance.

While the relatively poor model performance on the RAVEN stressor likely stems from its weaker elicited physiological responses (AVNN reactivity is displayed in Figure 2), the similarly low performance on SSST is more surprising. One possible explanation is that SSST elicits a fundamentally different physiological response compared with the other mental stressors, which could account for the poor generalization of the ML models to the SSST stressor observed in Table 3. To test this hypothesis, we retrained our LR and XGBoost classifiers separately on each stressor, that is, training and testing exclusively on one condition at a time. Multimedia Appendix 14 summarizes these per-stressor results, showing that both models outperform random BLs across all mental stressors. Notably, SSST shows marked improvement: despite both models struggling to detect it in earlier analyses, it now achieves an AUROC of 0.73 for LR and 0.76 for XGBoost, comparable to other mental stressors. This indicates that the SSST task elicits detectable MS responses. However, when SSST is excluded during training, AUROC drops sharply when tested on it (Table 3). Together, this indicates that while our ML models can learn to discriminate between SSST and nonstress when trained on SSST stressors alone, the patterns that the ML models learn from other mental stressors do not generalize to SSST. Interestingly, a similar pattern is also observed for the RAVEN stressor, further supporting this interpretation.

### Discrimination From Physical Activity

Given that MS can elicit ECG responses similar to those induced by physical activity (Figure 1), our ML models may conflate metabolic demand with MS. We therefore examined condition-specific sensitivity and specificity for light (LPA) and MPA (at 1000 sampling rates, 55 features).

The XGBoost model achieved good sensitivity for MS (0.800; 95% CI 0.698‐0.889), with the LR also yielding acceptable performance (0.772; 95% CI 0.656‐0.877). For the LPA, XGBoost achieved a specificity of 0.787 (95% CI 0.662‐0.903), whereas LR performed comparably, with a bootstrapped mean specificity of 0.794 (95% CI 0.685‐0.895). However, our models performed worse for MPA (LR: 0.444, 95% CI 0.314‐0.576; XGBoost: 0.418, 95% CI 0.299‐0.542), suggesting that even with a comprehensive feature set of 55 features, distinguishing stress-induced ECG responses from those elicited by MPA remains challenging.

## Discussion

### Principal Results

We developed ML models to distinguish MS from a composite nonstress BL encompassing various everyday activities (eg, walking at a normal pace, recovering from MS, or vacuum cleaning). Using 55 features derived from the ECG signal, XGBoost achieved an AUROC of 0.741 and AUPRC of 0.706, while LR performed comparably (AUROC: 0.724; AUPRC: 0.692) at 1000 Hz. Both models achieved high sensitivity (XGBoost: 0.800; LR: 0.782) but low specificity (XGBoost: 0.512; LR: 0.509), with this pattern being particularly pronounced for MPA. Our results, therefore, highlight a fundamental challenge in distinguishing stress-induced cardiac responses from those driven by physical exertion when relying solely on ECG features. The ML models used in our study also demonstrated robustness to ECG signal downsampling, retaining more than 93% of performance using only 10 features.

While the nonlinear model (XGBoost) numerically outperformed our linear model (LR), the performance differences were small (<0.02 difference in AUROC and AUPRC at 1000 Hz), suggesting that complex nonlinear models offer only marginal benefits relative to linear BLs. One potential explanation is the so-called Rashomon effect [80], which posits that there exist many equally well-performing models for specific datasets—a phenomenon often observed in high-stakes applications and tabular datasets [81]. High outcome variability, likely present in our dataset, as physiological responses to mental stressors are both participant- and stressor-specific (Figure 2 displays the HRV response), increases the likelihood that a simple model will perform on par with a more complex one [82]. The fact that we do not apply time-series models to the raw ECG dataset using, for instance, deep learning approaches and instead work with tabular data also likely further contributes to this phenomenon.

Our findings reveal a clear trade-off between window size and model performance, as increasing the window size led to model improvements. This can be explained by more reliable features, as shortening the window size has been shown to affect the quality of HRV features [25]. Deep learning approaches, such as convolutional or recurrent neural networks, can partially overcome this trade-off, as they learn feature representations directly from the ECG signals and have been shown to achieve promising performance, even using shorter window sizes than those considered in our study [83,84]. However, these models are often considered “black boxes” and typically require more data for training than the ML models considered in our study. Although recent advances in time-series explainability methods, such as those by Crabbé and Schaar [85] and Enguehard [86], have been proposed to overcome the challenge of interpreting deep learning time-series models, these approaches typically highlight only key segments in the time-series signal driving the model prediction. However, interpreting raw time series is challenging for humans. In contrast, our ML models use features based on well-studied physiological constructs, thereby facilitating the interpretation of model predictions using methods such as SHAP.

The feature importance analysis revealed the importance of fuzzy entropy for discriminating MS responses from other nonstress states. This finding aligns with prior work, which indicates that entropy-based measures, capturing the irregularity of the ECG signal, increase during periods of MS [87]. Notably, 2 HR fragmentation indices, PSS and IALS, also emerged as important features, despite being less commonly used in ML models for detecting MS. Our findings further highlight the importance of frequency-based features, consistent with a comprehensive review [28] covering 37 studies. In their studies, the authors identified changes in the low- and high-frequency components of the ECG signal as the most reported marker of MS.

Additionally, our analysis identified the VHF (as opposed to the UHF) band as an important predictor. Although the physiological origin of the VHF band is less well understood than that of the LF or HF bands, its predictive value likely reflects stress-induced hyperventilation, a well-documented physiological response to various stressors [35-37]. While the VHF band remained an important feature for distinguishing MS from a seated BL (Multimedia Appendix 9; Figures 3-5), suggesting a physiological contribution under sedentary conditions, we acknowledge that this band might also represent motion artifacts or electromyogram noise during active conditions. The low specificity observed during MPA may partially reflect this confound: If VHF power increases during physical activity, due to motion artifacts rather than vagal modulation, the model may misclassify physical exertion as MS. Further research on this band under stress is needed, particularly using accelerometry or respiratory measurements to distinguish stress-related changes from motion artifacts.

Our leave-one-stressor-out experiments demonstrated that our ML models could generalize to most held-out stressors, though performance varied by stressor. While SSST and Raven proved to be the most difficult stressors to recognize, the opposite was observed for TA: both models detected MS evoked by TA and its repeat condition, even when they had never been trained on it. This suggests that the TA stressor produces a strong and distinct physiological ECG response that can be easily distinguished from nonstress states. Whether this is related to differential psychological processes engaged by these tasks is an open question. The TA evokes frustration and a fear of punishment, whereas the SSST is social-evaluative and invokes a fear of exclusion, similar to Raven and mental arithmetic tasks, which also involve ego threats. Building on this, our findings imply that robust, generalizable models require training on a diverse set of various distinct mental stressors, or, when constrained, focus on mental stressors that evoke a strong physiological response, such as TA.

Although our results demonstrate that the ML models can detect MS with high sensitivity, distinguishing stress-evoked ECG responses from those induced by MPA remains especially challenging (specificity LR: 0.444; XGBoost: 0.418), even with a comprehensive set of 55 handcrafted features. Because deep learning models can learn more nuanced temporal ECG patterns, a possible path forward is to apply time-series deep learning directly to raw ECG data. Moreover, incorporating additional sensing modalities (eg, accelerometer or electrodermal activity) may help the model separate cardiac responses driven by metabolic demand from those driven by psychological stress. We therefore consider deep learning–based multimodal approaches an important direction for future research.

Importantly, our results suggest potential for translation to photoplethysmography (PPG)-based settings, as the most predictive features in our ML models are derived from beat-to-beat dynamics, specifically, the R-peaks and interbeat intervals, rather than ECG waveform morphology (eg, T-wave amplitude). Notably, the XGBoost model maintains most of its performance with 10 features (AUROC: 0.729) or 5 features (AUROC: 0.716; Figure 6). These feature subsets consist of PPG-compatible features (HR statistics and frequency-domain features; Multimedia Appendix 11), thereby supporting the possibility that our results may generalize to PPG signals. Consistent with this potential, Taoum et al [88] demonstrated that HRV metrics derived from PPG agreed with the ECG counterpart for 37 out of 48 features for recordings of 5 minutes and 30 seconds, and further for 16 features in 1-minute and 30-second windows. However, since we train on 30-second ECG epochs, future work must determine whether PPG devices can reliably extract those intervals in similarly short windows. Encouragingly, our models remain robust even when the ECG is downsampled to 125 Hz, supporting deployment on lower-resolution PPG wearables. Yet, PPG signals are more susceptible to motion artifacts than research-grade ECG [89], which may challenge the direct transfer of our feature pipeline. Given these considerations, validating and adapting our models for real-world PPG recordings, particularly under movement conditions and within 30-second windows, is therefore an important direction for future research and essential before our approach can be deployed on PPG-based wearables.

### Comparison With Prior Work

#### Dataset and Experimental Paradigms

MS detection has gained significant traction in recent years (Gedam and Paul [90] and Sharma and Gedeon [91] provide comprehensive reviews, Can et al [92] provides stress detection in daily life scenarios, and Pataca et al [93] provides wearable-based approaches), driven in part by the collection of large, multimodal datasets, some of which are publicly available, such as SWELL knowledge work [14], wearable stress and affect detection [13], StressID [61], or ForDigitStress [62]. While these datasets vary widely in modality, stressor type, and experimental setup, the majority rely on laboratory settings, inducing stress using the Trier Social Stress Test [94], variants of the Stroop task [95], or mental arithmetic challenges [63], all of which are effective methods for inducing acute stress [96].

Even though these datasets have significantly advanced the field, our experimental protocol and its associated dataset offer 2 distinct advantages compared with prior work.

First, our dataset includes 127 participants, which exceeds the sample sizes of most comparable ECG-based stress datasets [13,14,61]. Our relatively large sample size helps to enhance the robustness and generalizability of our findings, especially given the high between-individual variability in physiological stress responses [26,63,97] (also present in our study; see Figure 2). The small number of participants has also been noted as a limitation of prior work in a systematic review [98]. Notably, Smets et al [99] include 1002 participants; however, their study relies on an ambulatory design that uses self-reported stress via ecological momentary assessment. In contrast, our controlled laboratory protocol induces MS through standardized tasks and captures objective ECG measurements, which can be continuously observed.

Second, our experimental design incorporates 4 distinct mental stressors—2 of which are repeated—as well as a diverse set of nonstress conditions, including seated BL, recovery, or common everyday activities such as walking at a normal pace. This comprehensive experimental setup allows us to address a more ecologically valid and challenging classification problem: evaluating whether mental-stress-specific ECG patterns can be distinguished not only from rest, but also from other non-resting states.

#### ML Performance and Analysis

Most ECG-based stress-detection studies frame the problem as distinguishing MS from a seated, nonstress BL [13,61,62,100]. In contrast, Sun et al [63] achieved 80.9% between-individual accuracy in 20 participants by using an activity-aware, multimodal classifier, therefore combining ECG, accelerometer, and galvanic skin response to detect MS while sitting, standing, and walking. When the accelerometer input was removed, their performance dropped substantially, highlighting the importance of tracking the motion context. Although our ECG-only model does not match the multimodal performance reported by Sun et al [63], it is worth noting that their approach relies on a 60-second window. In contrast, our study uses a 30-second window, which, as stated above, reduces model performance but enables faster detection. Moreover, our laboratory setup encompasses a broader range of mental stressors and leverages an expanded ECG feature set, highlighting the potential of feature-rich, single-sensor models.

Previous work has often simplified stress detection to distinguishing MS from a seated BL [13,61]. In contrast, our classification task differentiates stress from a composite set of nonstressful everyday activities (eg, walking at a normal pace, poststress recovery, and vacuum cleaning), yielding a more ecologically valid scenario in which people naturally move and recover throughout their day. However, this setup makes our classification task more challenging, and consequently, performance is lower than in studies limited to seated nonstress conditions. When we rerun our models on the classic stress versus seated BL task, AUROC and AUPRC both increase markedly (Multimedia Appendix 12), confirming that the drop in metrics stems from the more demanding classification problem.

Beyond the expanded task, our approach also leverages a rich feature space of 55 time-, frequency-, nonlinear, and morphological ECG features, exceeding the number of ECG predictors used in other studies [13,61,101]. We also include and investigate less commonly considered features, such as HR fragmentation indices, which proved to be of added value according to our feature importance analysis (PSS and IALS). To the best of our knowledge, no previous work has evaluated the impact of aggressive downsampling on downstream model performance. We show that reducing the sampling rate from 1000 to 125 Hz does not degrade performance. While our models were robust to sampling rates, the choice of the window size affected model performance: increasing the window size from 30 to 60 s led to notable performance improvements for both ML models. Consequently, our findings provide valuable insights into the effects of sampling rate and window size choices for MS detection, thereby informing the design of models for resource-constrained wearables.

#### Model Generalization Across Mental Stressors

Most mental-stress classification studies evaluate models only on the same stressors used during training, while far fewer assess generalization to unseen stressors—a critical capability for real-world deployment. Prior work often evaluates generalizability by training on one dataset and testing on another [17-19,101]. While informative, such cross-dataset evaluations conflate multiple sources of variation—individual demographics, sensor hardware, and stressor types—making it challenging to attribute performance drops to any specific factor. In contrast, our leave-one-stressor-out evaluation isolates stressor novelty, enabling a more targeted assessment of a model’s ability to detect MS under previously unseen conditions. As our leave-one-out-stressor experiments demonstrated, both LR and XGBoost could generalize to novel stressors, indicating the feasibility of ML models to adapt to unseen mental stimuli.

Taken together, our work advances MS detection from several perspectives. First, by leveraging an ECG dataset from 127 participants across 6 mental stressors and a diverse set of nonstress activities, our work addresses a more challenging, ecologically valid task: separating MS from a composite nonstress set that includes everyday activities, for which we demonstrate acceptable performance. Second, we demonstrate that unimodal ML ECG classifiers using 55 features are robust against downsampling from 1000 to 125 Hz, maintaining a performance of over 95% with just 10 features. This highlights the feasibility of lightweight and interpretable models for wearable deployment. Third, through our leave-one-stressor-out evaluation, we provide valuable insights into model generalization to unseen stressors, isolating the challenge of stressor novelty without confounds from demographic or hardware differences.

### Limitations

Despite the results and insights presented in the paper, a few limitations warrant attention.

First, our cohort comprises Dutch-speaking young working adults (aged 18‐48 years). While our analytical framework is broadly applicable, physiological stress responses are highly individual and may vary across age groups, cultures, and health statuses.

Additionally, activity patterns might change over the lifespan [102], further limiting generalizability. Importantly, our results do not directly generalize to older populations, given that HRV, HR reserve, and other time-domain features generally decline with age [103], potentially making the classification problem more difficult. Given that distinguishing MS from cardiac responses evoked by physical activity proved very challenging despite our comparable young cohort, we anticipate that this challenge will persist (or even become more complicated) when considering an older population. An interesting direction for future work is therefore to validate our models in more diverse populations, such as older adults or non-Western cohorts, to ensure robustness and generalizability of our findings.

Second, we used global z-standardization across the training set to enable generalization to unseen users without requiring per-user calibration data. While this approach is attractive for real-world deployment, it does not account for the law of initial values [104], that is, it ignores interindividual variability in physiological responses to mental stressors present in our study (Figure 2). Our global normalization scheme may therefore bias model predictions against specific physiological phenotypes, for instance, by increasing false-positive rates among individuals with elevated resting HR or reduced BL HRV. Future work could examine the extent to which global versus individual-aware normalization strategies systematically affect downstream model performances across different BL physiological characteristics.

Third, we rely solely on task labels rather than participants’ self-reports to assign MS conditions, which prevents us from confirming that every labeled stress window corresponds to actual perceived stress. Incorporating self-assessments of perceived stress could have therefore reduced label noise, particularly given the pronounced interindividual variability in physiological responses (Figure 2 and Multimedia Appendix 3), which may partly account for the observed failure to generalize to the SSST stressor. Furthermore, instead of treating stress as a binary variable (stress or nonstress), one could use perceived ratings to classify the specific level or intensity of perceived MS [105].

Fourth, a related limitation of this study concerns the labeling of recovery periods. Physiological recovery from stress is gradual rather than instantaneous, suggesting that segments labeled “non-stress” during recovery periods likely retain residual stress physiology, constituting class-dependent label noise [106]. Empirically, label noise has been shown to adversely affect model performance across various ML approaches, including deep learning methods [107-109]. Our sensitivity analysis supports this observation: excluding recovery periods improved AUPRC for LR and XGBoost (see “Model Performance under Simplified Conditions”). We retained recovery periods as nonstress for comparability with prior stress-detection literature, which commonly assigns labels based on the protocol, and to provide conservative performance estimates. Yet, an alternative approach could either treat recovery as a buffer period excluded from training and evaluation or apply methods such as importance reweighting [110] or weighted surrogate loss [111].

Fifth, while our study uses ECG data to detect MS and downsamples the signal to mimic the hardware constraints of consumer-grade devices, such wearables typically rely on PPG. The most predictive features in our models are derived from R-peaks and beat-to-beat dynamics, which can also be extracted from PPG, suggesting that our approach may be transferable. However, this hypothesis remains to be validated empirically. Future work should therefore assess the extent to which our findings can be generalized to PPG-based settings.

Last, the MS tasks in our experimental setup were performed while participants remained seated. In everyday life, however, individuals often experience MS while standing or moving, for example, a teacher navigating a lively classroom. Future work should therefore extend the evaluation to simultaneous stress-and-activity scenarios to fully validate ambulatory MS detection, as done by Sun et al [63], Hosseini et al [112], and Kaczor et al [113].

### Conclusions

This study evaluated ML models for distinguishing MS from routine physical activities using a single-sensor ECG. Both LR and XGBoost achieved acceptable discriminative performance (AUROC >0.7), with XGBoost providing only marginal benefits over the linear BL (*Δ*<0.02), suggesting that simple, interpretable models can perform competitively. Performance remained robust when downsampling from 1000 to 125 Hz and reducing the feature set to 10, thereby supporting lightweight deployment. However, our findings reveal a critical limitation of single-sensor ECG approaches: although both models detected MS with acceptable to good sensitivity (XGBoost: 0.800; LR: 0.782), specificity was low (XGBoost: 0.512; LR: 0.509), particularly for MPA. This indicates that stress-induced cardiac responses cannot be reliably distinguished from those driven by physical exertion using ECG features alone. Generalization to unseen stressors was also stressor-dependent, with models performing well above chance for most stressors but near chance for the social-evaluative stimulus (SSST). Future studies should validate our findings in ambulatory settings where MS and physical activity can co-occur and explore both multimodal approaches and the use of PPG via wearables as a practical alternative to ECG for continuous stress monitoring.

## Supplementary material

10.2196/80450 Multimedia Appendix 1 Overview of features.

10.2196/80450 Multimedia Appendix 2 Hyperparameter settings.

10.2196/80450 Multimedia Appendix 3 Boxplots positive and negative affect.

10.2196/80450 Multimedia Appendix 4 Area under the precision-recall curve classification performance.

10.2196/80450 Multimedia Appendix 5 CIs for performance differences between XGBoost and LR.

10.2196/80450 Multimedia Appendix 6 Additional classification performance results.

10.2196/80450 Multimedia Appendix 7 Seated baseline classification performance.

10.2196/80450 Multimedia Appendix 8 Time window analysis.

10.2196/80450 Multimedia Appendix 9 Model explainability.

10.2196/80450 Multimedia Appendix 10 Feature parsimony.

10.2196/80450 Multimedia Appendix 11 Overview of selected features.

10.2196/80450 Multimedia Appendix 12 Generalization to unknown stressors.

10.2196/80450 Multimedia Appendix 13 Stratified stressor analysis.

10.2196/80450 Multimedia Appendix 14 Stratified stressor analysis - single sensor training.

## Abbreviations

ANS: autonomic nervous system
AUPRC: area under the precision-recall curve
AUROC: area under the receiver operating characteristic
AVNN: average normal-to-normal
BL: baseline
BPM: beats per minute
ECG: electrocardiogram
HF: high frequency
HR: heart rate
HRA: heart rate asymmetry
HRV: heart rate variability
Hz: hertz
IALS: inverse average length of segments
LPA: low physical activity
LR: logistic regression
ML: machine learning
MMA: modified moving average
MPA: moderate physical activity
MS: mental stress
NN: normal-to-normal
PASAT: paced auditory serial addition task
PPG: photoplethysmography
PSS: percentage of short segments
RAVEN: Raven’s Progressive Matrices
RF: random forest
RMSSD: root-mean-square of successive differences
SDNN: SD of the NN intervals
SHAP: Shapley additive explanation
SMOTE: synthetic minority oversampling technique
SQI: signal quality index
SSST: sing-a-song-stress test
TA: tone avoidance
TRIPOD+AI: Transparent Reporting of a Multivariable Prediction Model for Individual Prognosis or Diagnosis–Artificial Intelligence
TWA: T-wave alternans
UHF: ultra-high frequency
VHF: very high frequency
XGBoost: extreme gradient boosting
