# Supplementary material for: Electrocardiogram-Based Mental Stress Detection Amid Everyday Activities Using Machine Learning: Model Development and Validation Study
Source: J Med Internet Res. 2026 Apr 7;28:e80450. doi: 10.2196/80450 (PMC13055957; doi:10.2196/80450)
Supplement: Multimedia Appendix 1 [file jmir-v28-e80450-s001.pdf]

Overview of features

In this section, we give an overview of features extracted from the preprocessed Electrocardiogram (ECG) signal. Overall, 55 features were considered in our study.

Time-domain Features

Heart rate: Mean, SD, min, max;
RMSSD: Root mean square of successive differences (RMSSD); **AVNN:** Average value of normal-to-normal (NN) intervals; **SDNN:** SD of NN intervals; **NN20:** Number of (normal-to-normal) NN intervals differing by more than 20 milliseconds (ms); **PNN20:** Percentage of consecutive NN intervals differing by more than 20ms;
IQRNN: Interquartile range NN intervals; **MADNN:** Median absolute difference NN intervals; **SDRMSSD:** SDNN divided by RMSSD; **NN50:** Number of consecutive NN intervals differing by more than 50ms; **PNN50:** Percentage of successive NN intervals differing by more than 50ms; **CVNN:** SDNN divided by AVNN**; CVSD:** RMSSD divided by AVNN;

Frequency-domain Features

High Frequency (HF) (0.15–0.40 Hertz): Mean, min, max, median, SD, power, entropy;
Very High Frequency (VHF) (0.40–0.50 Hertz): Mean, min, max, median, SD, power, entropy;
Ultra High Frequency (UHF) (0.50–1.00 Hertz): Mean, min, max, median, SD, power, entropy;
Relative Power: Relative power of respective frequency band to overall power across all frequency bands considered (HF, VHF, UHF);

Non-linear Features

Fuzzy Entropy; Approximate Entropy; SD1: The SD of the points along the identity line in a poincaré plot; **SD2:** The SD of the points perpendicular to the identity line in a poincaré plot; **SD1/SD2:** The ratio between SD1 and SD2; **W:** The average white vertical line length of a recurrence quantification analysis (RQA); **WMax:** Longest white vertical line length of a RQA; **WEn:** Entropy of white vertical line length of a RQA;
PSS: The complement of the percentage of NN intervals in acceleration or deceleration with three or more NN intervals [1]; **PIP**: Percentage of inflection points of the RR intervals series [1]. **IALS**: Inverse of average length of the acceleration and deceleration segments [1]. **PAS**:
Percentage of NN intervals in alternation segments [1];
DFA α1: Detrended fluctuation analysis (DFA) α1 represents short-term fluctuations. **Area index:** The ratio of the total area of points **above the line of identity (LI)** to the total area of all points in the Poincaré plot (except for the ones on the LI).

Morphology

TWA: The variation in amplitude of the T-wave (repolarization of the heart).

References

1. Costa MD, Davis RB, Goldberger AL. Heart rate fragmentation: a new approach to the analysis of cardiac interbeat interval dynamics. Front Physiol. May 2017;8. [doi: 10.3389/fphys.2017.00255]
