# Supplementary material for: Electrocardiogram-Based Mental Stress Detection Amid Everyday Activities Using Machine Learning: Model Development and Validation Study
Source: J Med Internet Res. 2026 Apr 7;28:e80450. doi: 10.2196/80450 (PMC13055957; doi:10.2196/80450)
Supplement: Multimedia Appendix 2 [file jmir-v28-e80450-s002.pdf]

Hyperparameter settings

In this section, we provide additional information on the hyperparameter search space used for the logistic regression (LR), extreme gradient boosting (XGBoost), and random forest (RF) models. As stated in the main text of our paper, hyperparameters for the machine learning models were optimized via Bayesian optimization using Gaussian processes (Optuna version 4.2.1) over 25 iterations.

Logistic Regression

A L2 penalty was used, and the maximum number of iterations was set to 2,000. The cost parameter C was optimized over the range between 0.01 and 1 on a log scale. The class weight hyperparameter was optimally chosen between all available options (class_weight, balanced, None).

The optimal parameters were found to be 0.017 for C and balanced class weighting.

Extreme Gradient Boosting

The number of estimators was optimized over the range 75 to 150, while the maximum depth was tuned for the range 2 to 4. The learning rate was optimized over the log range 0.005 to 0.05. For the regularization parameters alpha and lambda, the search space was between 15 and 25, respectively. For the subsample parameter, the hyperparameter value was optimized between 0.3 and 0.6, whereas for the colsample_bytree hyperparameter, the search space was restricted within the range of 0.3 to 0.7.

The optimal parameters were found to be 140 for the number of estimators, 4 for the maximum depth, 0.038 for the learning rate, 0.368 for the subsample, 0.576 for the colsample_bytree, 22.003 for the lambda regularization, and 16.257 for the alpha regularization.

Random Forest

The number of estimators was optimized over the range 50 to 300, while the maximum depth was tuned over 5 to 50. Both the minimum samples per split and the minimum samples per leaf were optimized over the range 5 to 25. The class weight hyperparameter was optimally chosen from all available options, including None.

The optimal parameters were found to be 198 for the number of estimators, 7 for the maximum depth, 17 for the minimum number of samples per split, and 8 for the minimum number of samples per leaf, with no specific class weights provided.
