# Supplementary material for: Electrocardiogram-Based Mental Stress Detection Amid Everyday Activities Using Machine Learning: Model Development and Validation Study
Source: J Med Internet Res. 2026 Apr 7;28:e80450. doi: 10.2196/80450 (PMC13055957; doi:10.2196/80450)

Boxplots positive and negative affect

Figure S1. Difference in positive affect during mental stress conditions compared to baseline affect while sitting. Positive affect was rated on a scale of 1 (not at all) to 7 (very) for the items "cheerful", "enthusiastic", "relaxed", and "content", with positive affect representing the average score across all items. Statistical significance was assessed using a paired t-test. Test statistics: PASAT: t_{126}=-13.25, P<.001; PASAT (repeat): t_{126}=-13.45, P<.001; RAVEN: t_{126}=-7.96, P<.001; SSST: t_{126}=-4.65, P<.001; TA: t_{126}=-7.71, P<.001; TA (repeat): t_{126}=-5.00, P<.001 for two-sided paired t-tests. PASAT: paced auditory serial addition task; RAVEN: Raven's progressive matrices; SSST: sing-a-song-stress test; TA: tone avoidance.

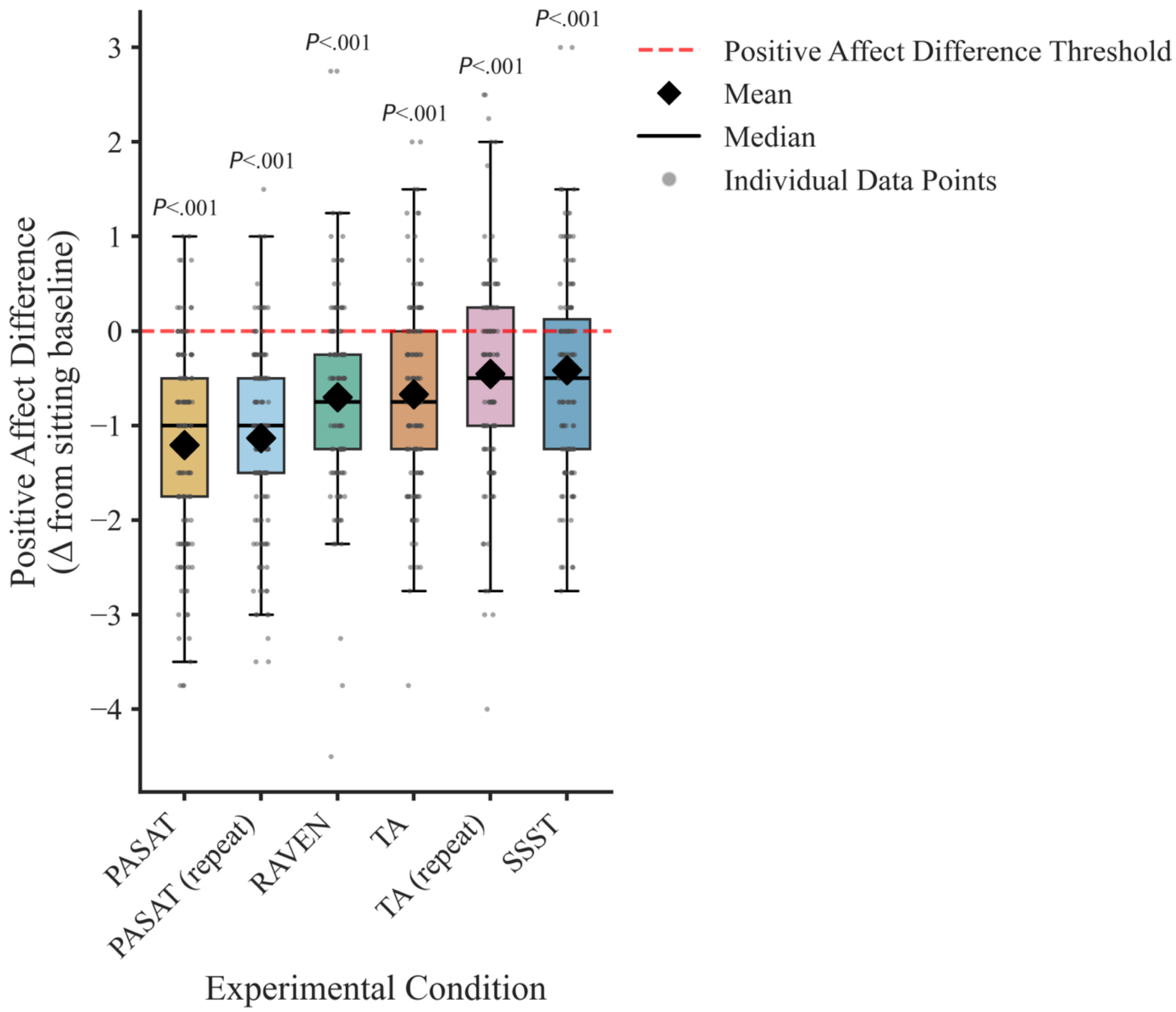

Figure S2. Difference in negative affect during mental stress conditions compared to baseline affect while sitting. Positive affect was rated on a scale of 1 (not at all) to 7 (very) for the items "insecure", "lonely", "anxious", "irritated", and "down", with negative affect representing the average score across all items. Statistical significance was assessed using a paired t-test. Test statistics: PASAT: t_{126}=-12.00, *P*<.001; PASAT (repeat): t_{126}=-6.57, *P*<.001; RAVEN: t_{126}=-6.09, *P*<.001; SSST: t_{126}=-6.67, *P*<.001; TA: t_{126}=-8.31, *P*<.001; TA (repeat): t_{126}=-3.21, *P*=.002 for two-sided paired t-tests. PASAT: paced auditory serial addition task; RAVEN: Raven's progressive matrices; SSST: sing-a-song-stress test; TA: tone avoidance.

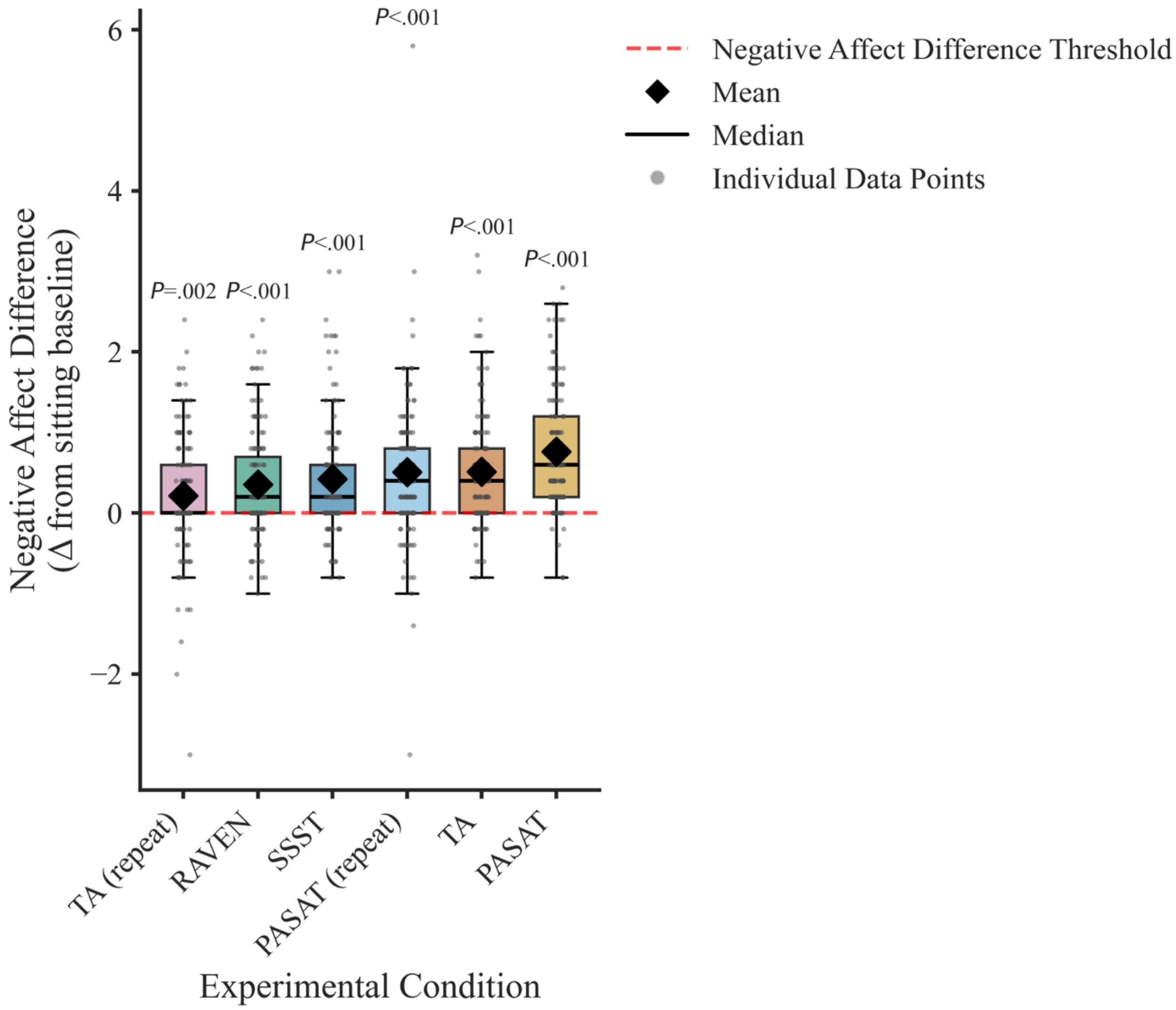

Supplement: Multimedia Appendix 3 [file jmir-v28-e80450-s003.pdf]
