# Supplementary material for: Electrocardiogram-Based Mental Stress Detection Amid Everyday Activities Using Machine Learning: Model Development and Validation Study
Source: J Med Internet Res. 2026 Apr 7;28:e80450. doi: 10.2196/80450 (PMC13055957; doi:10.2196/80450)

Area under the precision-recall curve classification performance

Figure S1. Performance comparison of LR and XGBoost for ECG-based mental stress classification across sampling rates (127 total participants, 26 test set participants). Points represent bootstrapped mean AUPRC score with 95% CIs (error bars) based on 2000 participant-level bootstrap samples. Models were trained on 55 features extracted from 30-second windows (10-second shift) using 60/20/20 (train/validation/test) splits at the individual level. Both models demonstrate robustness to downsampling from 1000 to 125 Hz. AUPRC: area under the precision-recall curve; CI: confidence interval; ECG: electrocardiogram; Hz: hertz; LR: logistic regression; XGBoost: extreme gradient boosting.

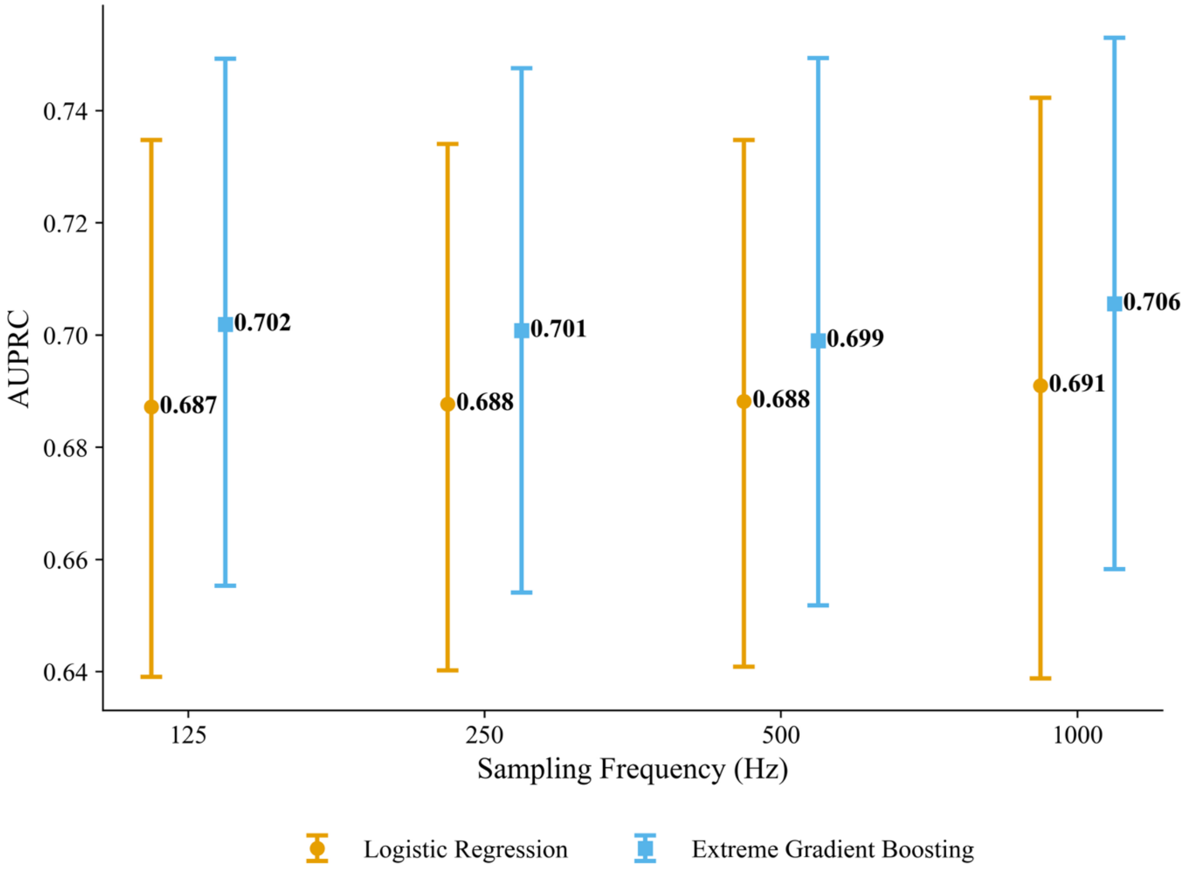

Supplement: Multimedia Appendix 4 [file jmir-v28-e80450-s004.pdf]
