# Supplementary material for: Electrocardiogram-Based Mental Stress Detection Amid Everyday Activities Using Machine Learning: Model Development and Validation Study
Source: J Med Internet Res. 2026 Apr 7;28:e80450. doi: 10.2196/80450 (PMC13055957; doi:10.2196/80450)
Supplement: Multimedia Appendix 5 [file jmir-v28-e80450-s005.pdf]

CIs for performance differences between XGBoost and LR

Table S1. Performance difference between XGBoost and LR for mental stress classification, evaluated on the held-out test set (127 total participants, 26 test set participants). Values show mean bootstrapped performance differences (XGBoost minus LR) with 99% CIs from 2000 participant-level bootstrap samples for AUROC and AUPRC across four ECG sampling rates (1000, 500, 250, and 125 Hz). Both models were independently trained using 60/20/20 (train/validation/test) splits at the individual level on 55 heart rate variability features extracted from 30-second windows (10-second shift). AUPRC: area under the precision-recall curve; AUROC: area under the receiver operating characteristic; CI: confidence interval; ECG: electrocardiogram; Hz: hertz; LR: logistic regression; XGBoost: extreme gradient boosting.

Frequency (Hertz)	99% CI Performance Difference (XGBoost – LR)	
	AUROC	AUPRC
1000	0.0171 (-0.0059 to 0.0362)	0.0146 (-0.0143 to 0.0445)
500	0.0096 (-0.0082 to 0.0268)	0.0108 (-0.0118 to 0.0320)
250	0.0112 (-0.0086 to 0.0296)	0.0130 (-0.0116 to 0.0369)
125	0.0119 (-0.0099 to 0.0319)	0.0147 (-0.0104 to 0.0357)

Table S2. Performance difference between XGBoost and LR for mental stress classification, evaluated on the held-out test set (127 total participants, 26 test set participants). Values show mean bootstrapped performance differences (XGBoost minus LR) with 95% CIs from 2000 participant-level bootstrap samples for AUROC and AUPRC across four ECG sampling rates (1000, 500, 250, and 125 Hz). Both models were independently trained using 60/20/20 (train/validation/test) splits at the individual level on 55 heart rate variability features extracted from 30-second windows (10-second shift). AUPRC: area under the precision-recall curve; AUROC: area under the receiver operating characteristic; CI: confidence interval; ECG: electrocardiogram; Hz: hertz; LR: logistic regression; XGBoost: extreme gradient boosting.

Frequency (Hertz)	95% CI Performance Difference (XGBoost – LR)	
	AUROC	AUPRC
1000	0.0171 (0.0010 to 0.0323)	0.0146 (-0.0070 to 0.0367)
500	0.0096 (-0.0038 to 0.0232)	0.0108 (-0.0065 to 0.0277)
250	0.0112 (-0.0038 to 0.0254)	0.0130 (-0.0047 to 0.0310)
125	0.0119 (-0.0035 to 0.0275)	0.0147 (-0.0037 to 0.0319)
