# Supplementary material for: Electrocardiogram-Based Mental Stress Detection Amid Everyday Activities Using Machine Learning: Model Development and Validation Study
Source: J Med Internet Res. 2026 Apr 7;28:e80450. doi: 10.2196/80450 (PMC13055957; doi:10.2196/80450)

Additional classification performance results

Figure S1. Performance comparison of LR, XGBoost, and RF for ECG-based mental stress classification across sampling rates (127 total participants, 26 test set participants). Points represent the bootstrapped mean AUROC scores with 95% CIs (error bars) based on 2000 participant-level bootstrap samples. Models were trained on 55 features extracted from 30-second windows (10-second shift) using 60/20/20 (train/validation/test) splits at the individual level. All models demonstrate robustness to downsampling from 1000 to 125 Hz. AUROC: area under the receiver operating characteristic; CI: confidence interval; ECG: electrocardiogram; Hz: hertz; LR: logistic regression; RF: random forest; XGBoost: extreme gradient boosting.

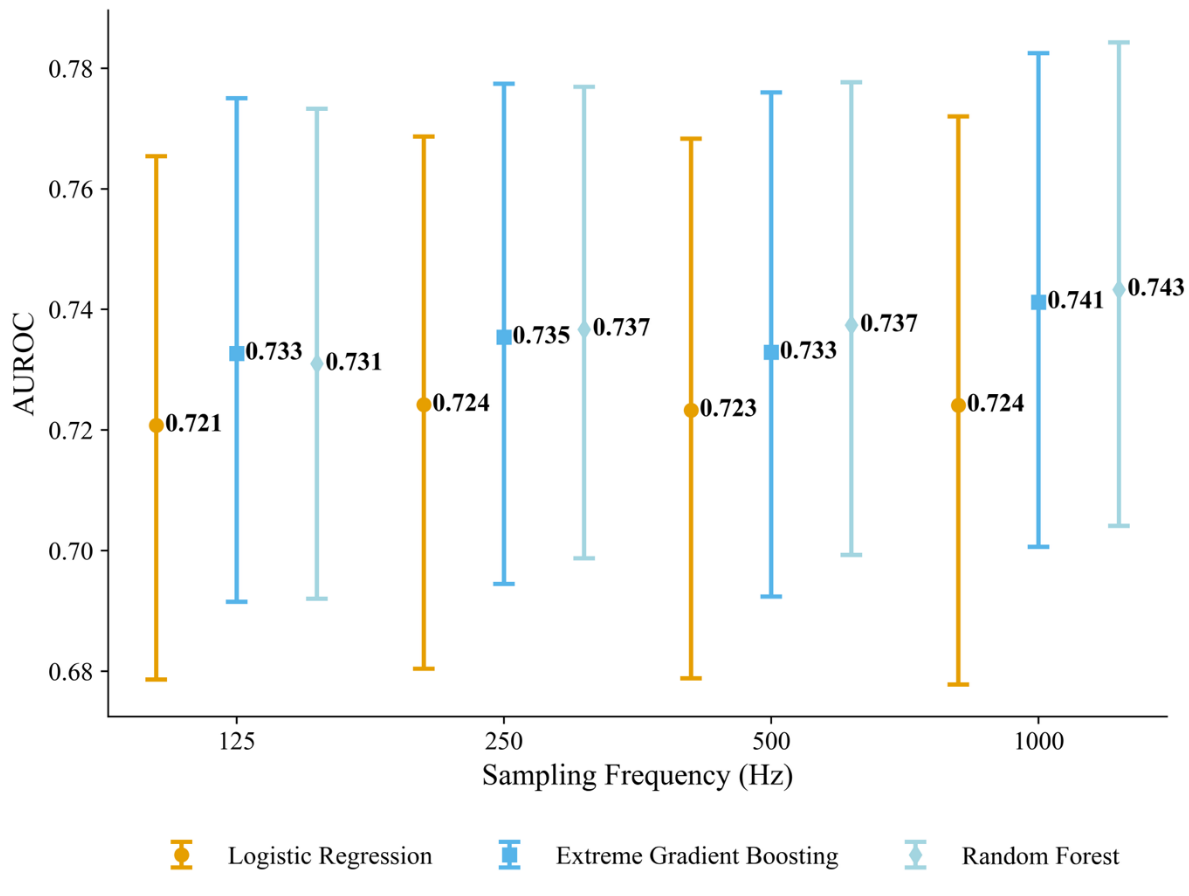

Figure S2. Performance comparison of LR, XGBoost, and RF for ECG-based mental stress classification across sampling rates (127 total participants, 26 test set participants). Points represent the bootstrapped mean AUPRC scores with 95% CIs (error bars) based on 2000 participant-level bootstrap samples. Models were trained on 55 features extracted from 30-second windows (10-second shift) using 60/20/20 (train/validation/test) splits at the individual level. All models demonstrate robustness to downsampling from 1000 to 125 Hz. AUPRC: area under the precision-recall curve; CI: confidence interval; ECG: electrocardiogram; Hz: hertz; LR: logistic regression; RF: random forest; XGBoost: extreme gradient boosting.

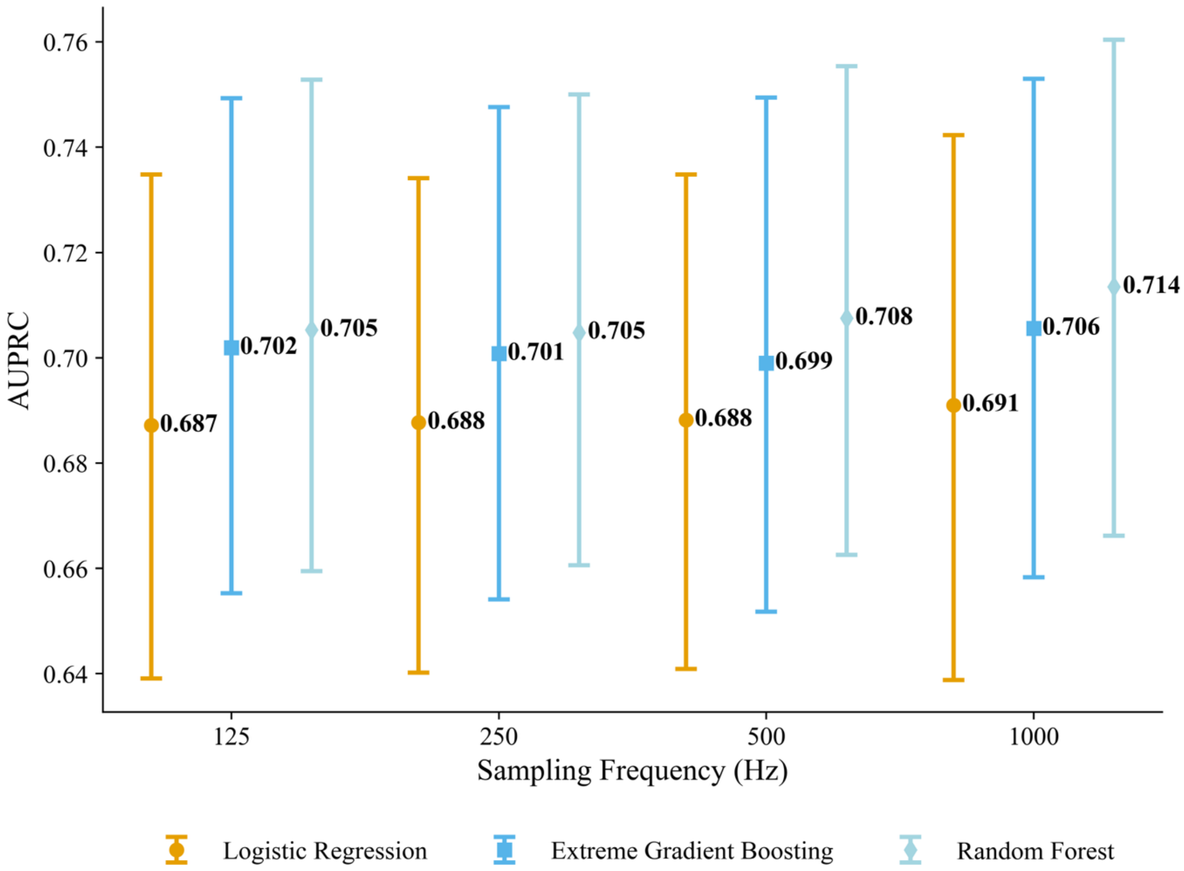

Figure S3. Performance comparison of LR, XGBoost, and RF for ECG-based mental stress classification across sampling rates (127 total participants, 26 test set participants). Points represent the bootstrapped mean F1-scores with 95% CIs (error bars) based on 2000 participant-level bootstrap samples. Models were trained on 55 features extracted from 30-second windows (10-second shift) using 60/20/20 (train/validation/test) splits at the individual level. All models demonstrate robustness to downsampling from 1000 to 125 Hz. CI: confidence interval; ECG: electrocardiogram; Hz: hertz; LR: logistic regression; RF: random forest; XGBoost: extreme gradient boosting.

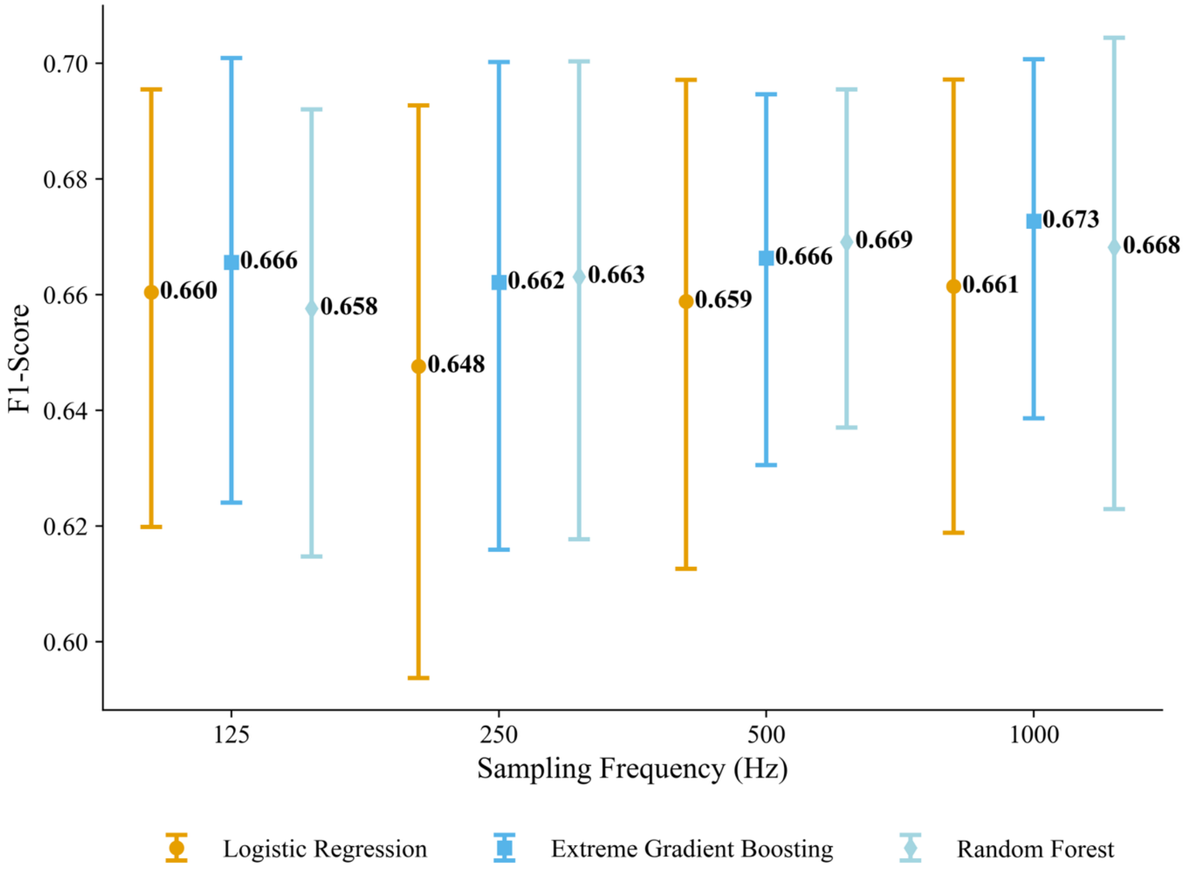

Figure S4. Performance comparison of LR, XGBoost, and RF for ECG-based mental stress classification across sampling rates (127 total participants, 26 test set participants). Points represent the bootstrapped mean sensitivity scores at the F1-maximizing threshold (determined on the validation set) with 95% CIs (error bars) based on 2000 participant-level bootstrap samples. Models were trained on 55 features extracted from 30-second windows (10-second shift) using 60/20/20 (train/validation/test) splits at the individual level. All models demonstrate robustness to downsampling from 1000 to 125 Hz. CI: confidence interval; ECG: electrocardiogram; Hz: hertz; LR: logistic regression; RF: random forest; XGBoost: extreme gradient boosting.

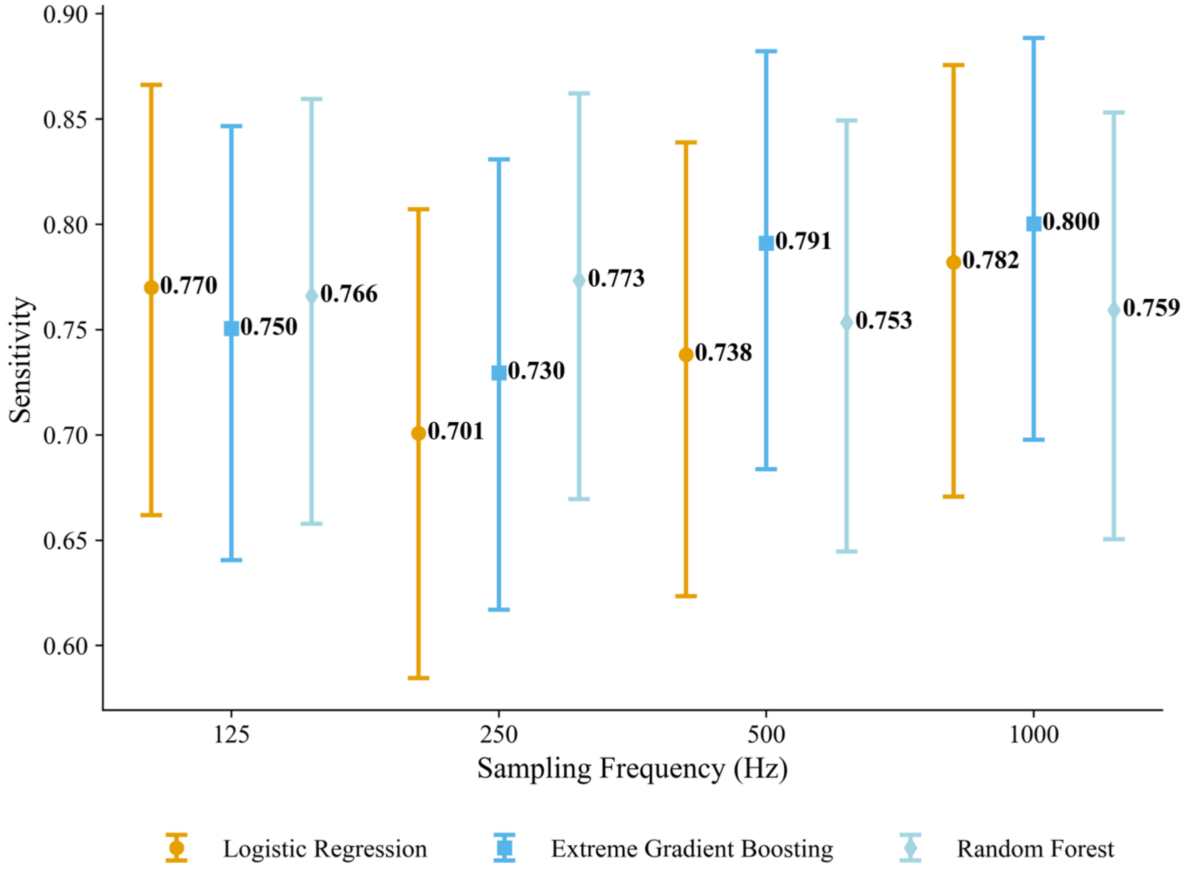

Figure S5. Performance comparison of LR, XGBoost, and RF for ECG-based mental stress classification across sampling rates (127 total participants, 26 test set participants). Points represent the bootstrapped mean specificity scores at the F1-maximizing threshold (determined on the validation set) with 95% CIs (error bars) based on 2000 participant-level bootstrap samples. Models were trained on 55 features extracted from 30-second windows (10-second shift) using 60/20/20 (train/validation/test) splits at the individual level. All models demonstrate robustness to downsampling from 1000 to 125 Hz. CI: confidence interval; ECG: electrocardiogram; Hz: hertz; LR: logistic regression; RF: random forest; XGBoost: extreme gradient boosting.

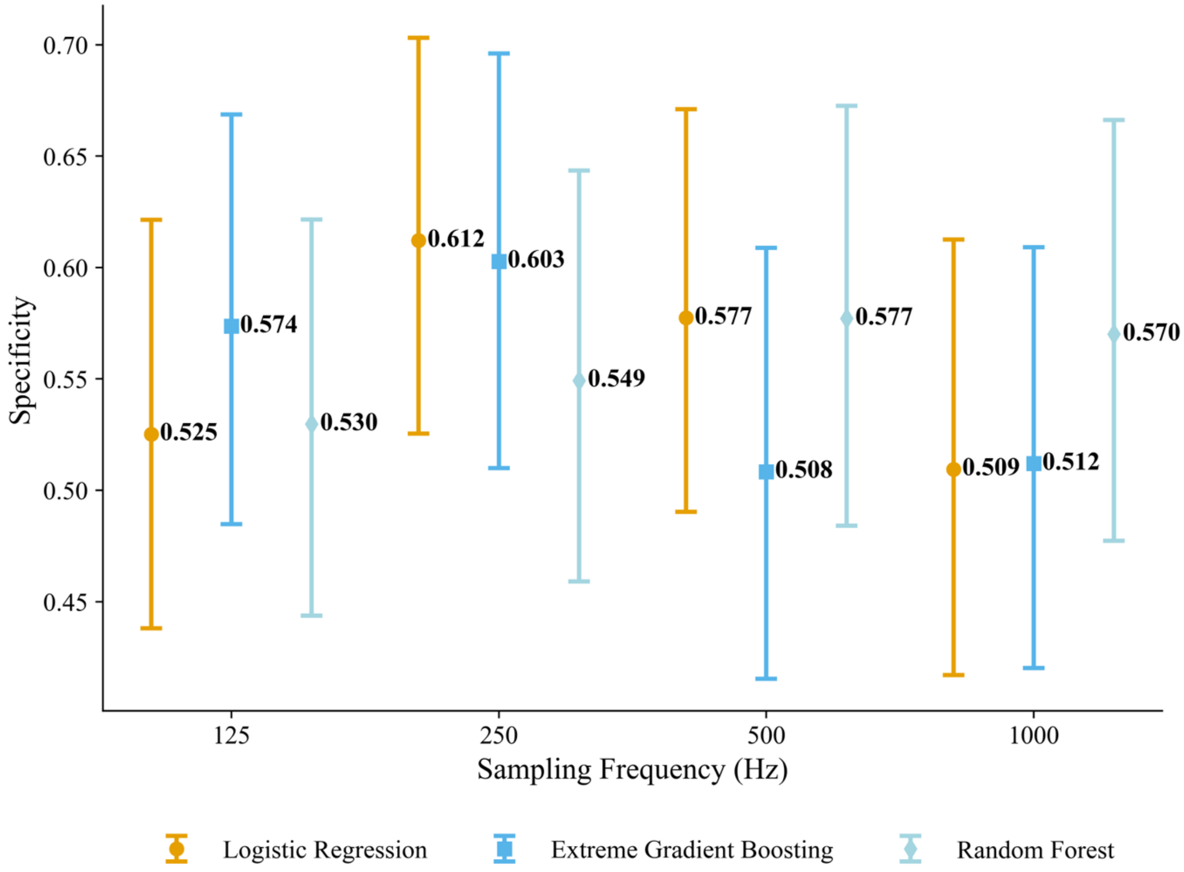

Supplement: Multimedia Appendix 6 [file jmir-v28-e80450-s006.pdf]
