# Supplementary material for: Electrocardiogram-Based Mental Stress Detection Amid Everyday Activities Using Machine Learning: Model Development and Validation Study
Source: J Med Internet Res. 2026 Apr 7;28:e80450. doi: 10.2196/80450 (PMC13055957; doi:10.2196/80450)

Seated baseline classification performance

Figure S1. Model performance across task complexities and sampling rates for mental stress classification (127 total participants, 26 test set participants). Points represent bootstrapped mean AUROC with 95% confidence intervals from 2000 participant-level bootstrap samples for LR and XGBoost. Solid lines show performance on the more challenging task: mental stress versus all non-stress conditions (seated baseline, light physical activity, and moderate physical activity). Dotted lines show performance on the simplified task, that is, mental stress versus a seated baseline (sitting and recovery periods). Models trained on 55 features from 30-second windows using 60/20/20 (train/validation/test) splits at the individual level. Both models show improved performance when physical activities are excluded from the non-stress class. AUROC: area under the receiver operating characteristic; LR: logistic regression; XGBoost: extreme gradient boosting.

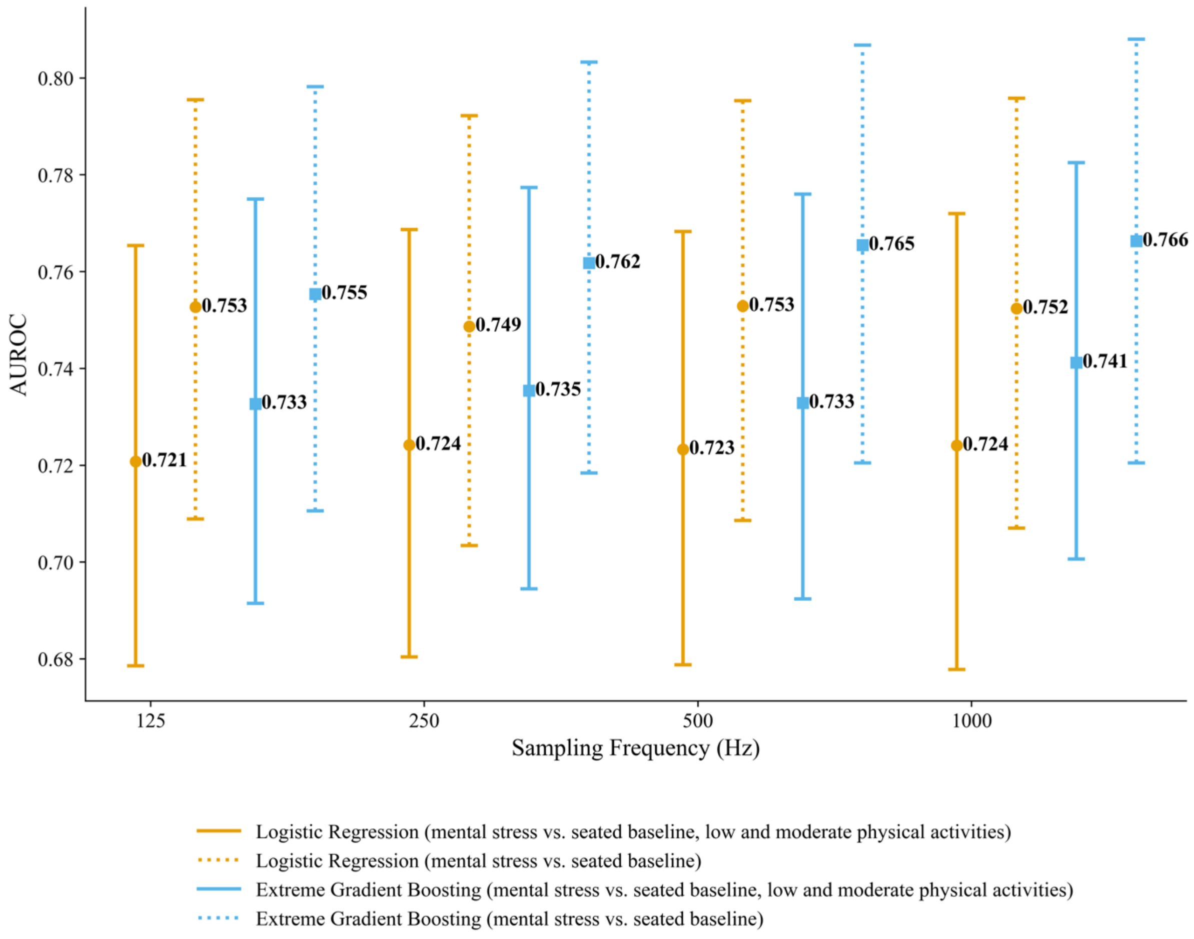

Figure S2. Model performance across task complexities and sampling rates for mental stress classification (127 total participants, 26 test set participants). Points represent bootstrapped mean AUPRC with 95% confidence intervals from 2000 participant-level bootstrap samples for LR and XGBoost. Solid lines show performance on the more challenging task: mental stress versus all non-stress conditions (seated baseline, light physical activity, and moderate physical activity). Dotted lines show performance on the simplified task, that is, mental stress versus a seated baseline (sitting and recovery periods). Models trained on 55 features from 30-second windows using 60/20/20 (train/validation/test) splits at the individual level. Both models show improved performance when physical activities are excluded from the non-stress class. AUPRC: area under the precision-recall curve; LR: logistic regression; XGBoost: extreme gradient boosting.

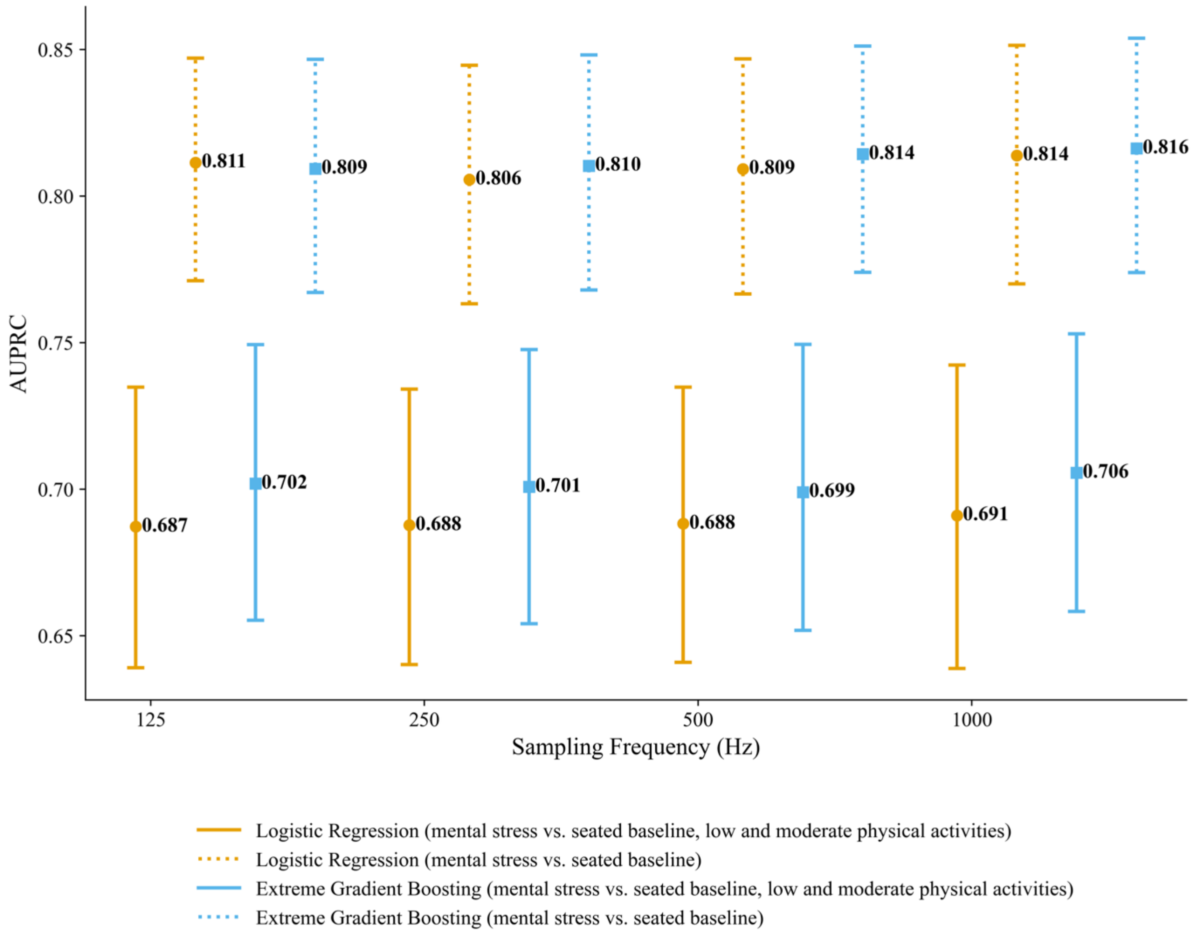

Supplement: Multimedia Appendix 7 [file jmir-v28-e80450-s007.pdf]
