# Supplementary material for: Electrocardiogram-Based Mental Stress Detection Amid Everyday Activities Using Machine Learning: Model Development and Validation Study
Source: J Med Internet Res. 2026 Apr 7;28:e80450. doi: 10.2196/80450 (PMC13055957; doi:10.2196/80450)

Time window analysis

Figure S1. Model performance across time window durations and varying sampling rates for mental stress classification (127 total participants, 26 test set participants). Bootstrapped mean AUROC with 95% confidence intervals based on 2000 participant-level bootstrap samples for LR and XGBoost trained with 30-second versus 60-second ECG windows. Models trained on 55 features using 60/20/20 (train/validation/test) splits at the individual level. Longer time windows improve performance, but at the expense of rapid mental stress detection. AUROC: area under the receiver operating characteristic; LR: logistic regression; XGBoost: extreme gradient boosting.

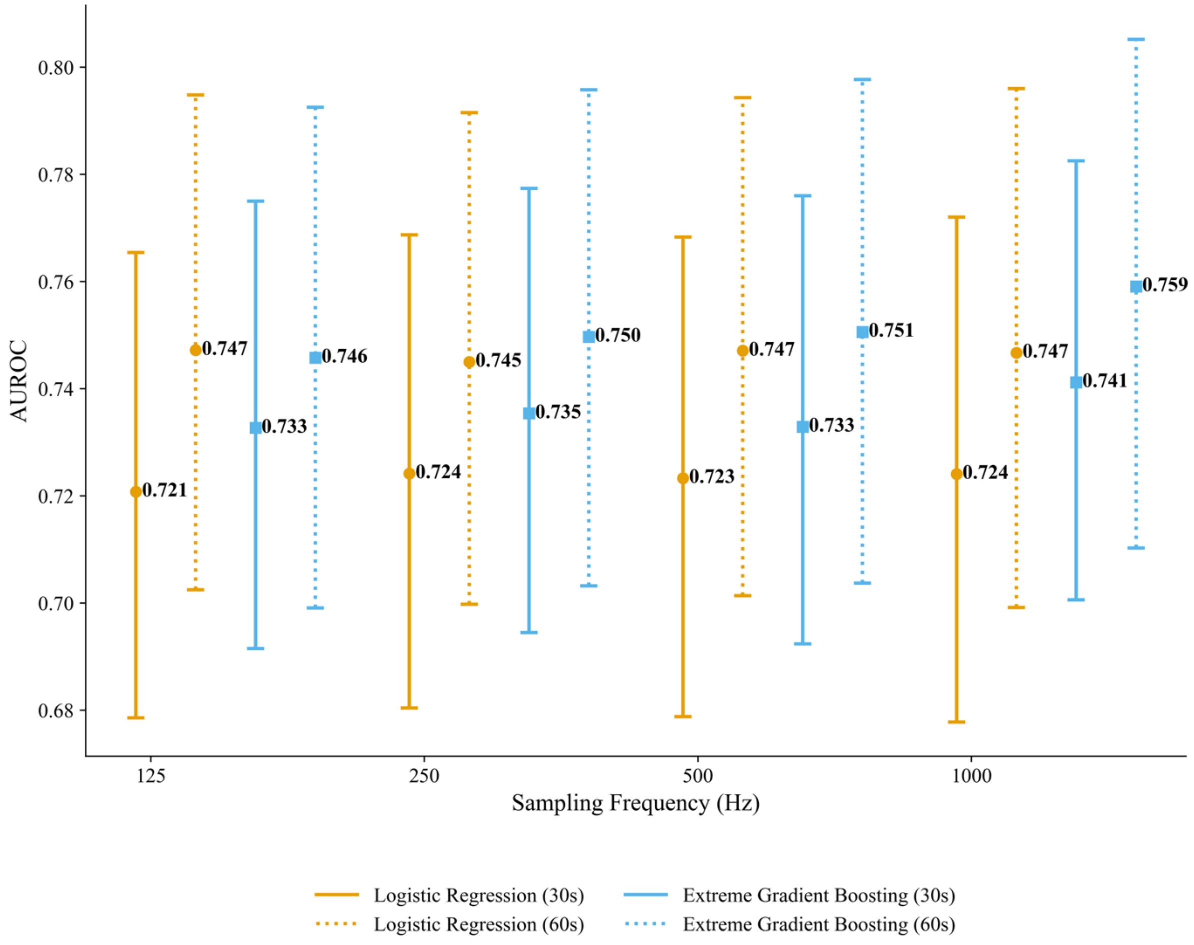

Figure S2. Model performance across time window durations and varying sampling rates for mental stress classification (127 total participants, 26 test set participants). Bootstrapped mean AUPRC with 95% confidence intervals based on 2000 participant-level bootstrap samples for LR and XGBoost trained with 30-second versus 60-second ECG windows. Models trained on 55 features using 60/20/20 (train/validation/test) splits at the individual level. Longer time windows improve performance, but at the expense of rapid mental stress detection. AUPRC: area under the precision-recall curve; LR: logistic regression; XGBoost: extreme gradient boosting.

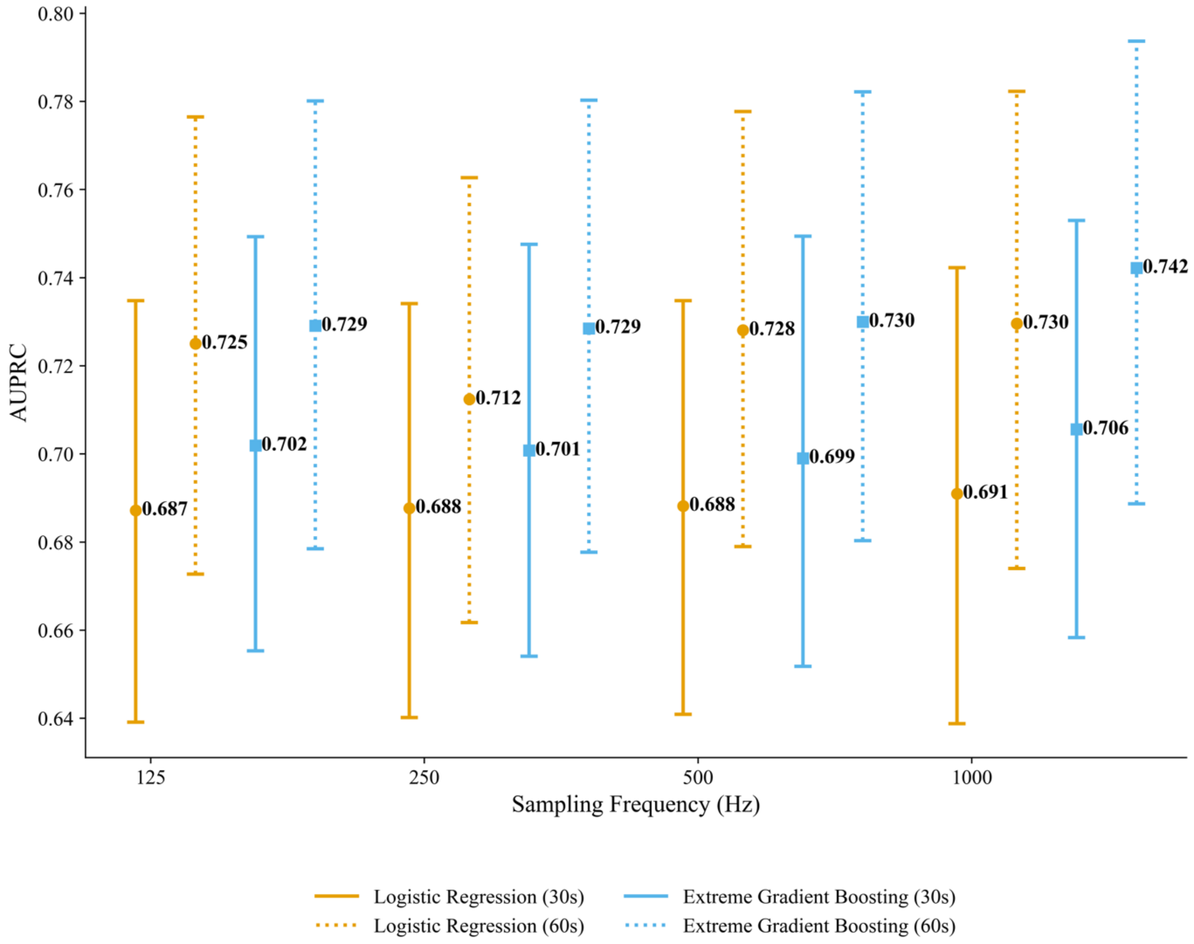

Supplement: Multimedia Appendix 8 [file jmir-v28-e80450-s008.pdf]
