# Supplementary material for: Electrocardiogram-Based Mental Stress Detection Amid Everyday Activities Using Machine Learning: Model Development and Validation Study
Source: J Med Internet Res. 2026 Apr 7;28:e80450. doi: 10.2196/80450 (PMC13055957; doi:10.2196/80450)

Model explainability

Figure S1. SHAP plot for the detection of mental stress versus no-mental-stress for the LR model. The top 10 most important features are presented in descending order of importance. Each dot represents a Shapley value for a specific instance and feature, with the color indicating the underlying feature value ranging from high (red) to low (blue). AVNN: average value of (NN) intervals; HF: high frequency (0.15–0.40 hertz); HR: heart rate; IALS: inverse of average length of the acceleration and deceleration segments; LR: logistic regression; PNN20: percentage of consecutive normal-to-normal (NN) intervals differing more than 20 milliseconds (ms); SD: standard deviation; SHAP: Shapley additive explanation; VHF: very high frequency (0.40–0.50 hertz); Wen: entropy of white vertical line length of a recurrence quantification analysis.

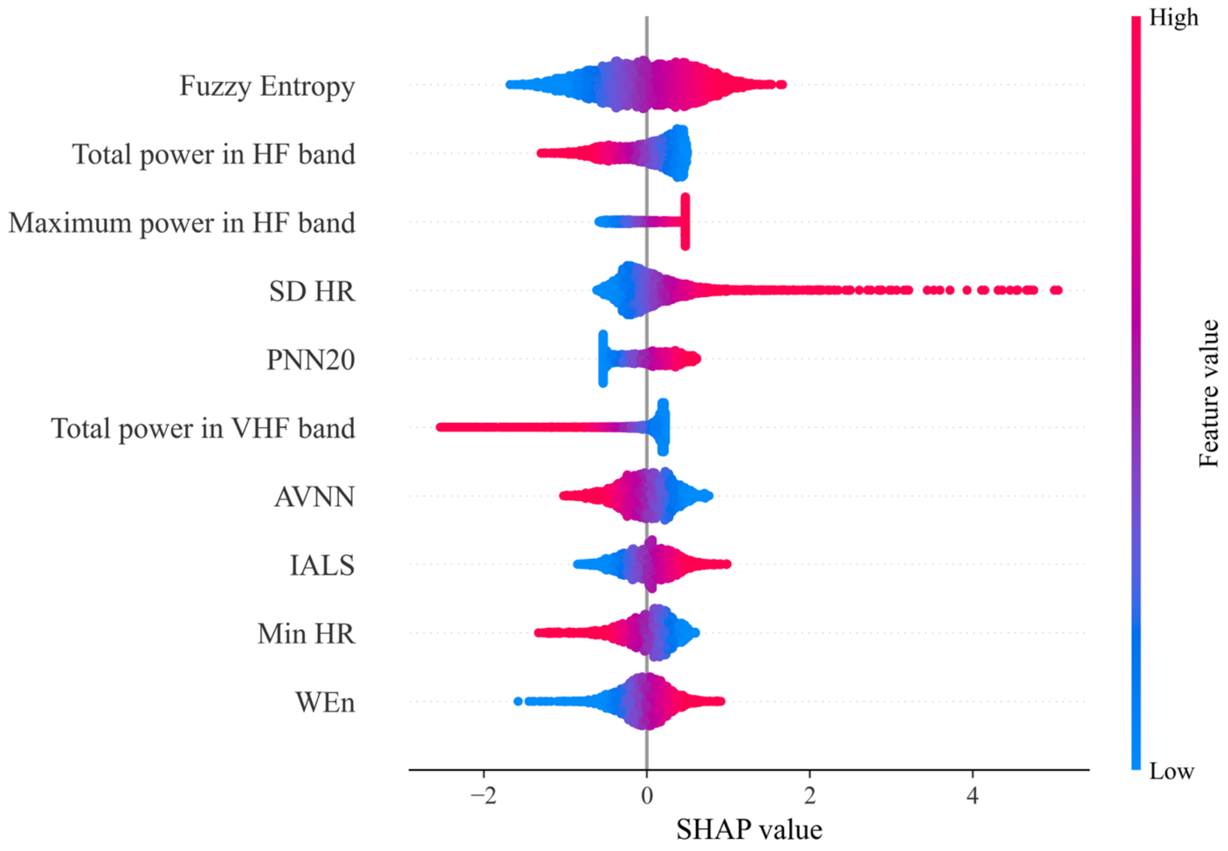

Figure S2. Top 10 features ranked by absolute coefficient magnitude in the LR model for mental stress versus non-stress classification. AVNN: average value of (NN) intervals; HF: high frequency (0.15–0.40 hertz); HR: heart rate; IALS: inverse of average length of the acceleration and deceleration segments; LR: logistic regression; PNN20: percentage consecutive normal-to-normal (NN) intervals differing by more than 20 milliseconds (ms); SD HR: standard deviation heart rate; VHF: very high frequency (0.40–0.50 hertz); Wen: entropy of white vertical line length of a recurrence quantification analysis.

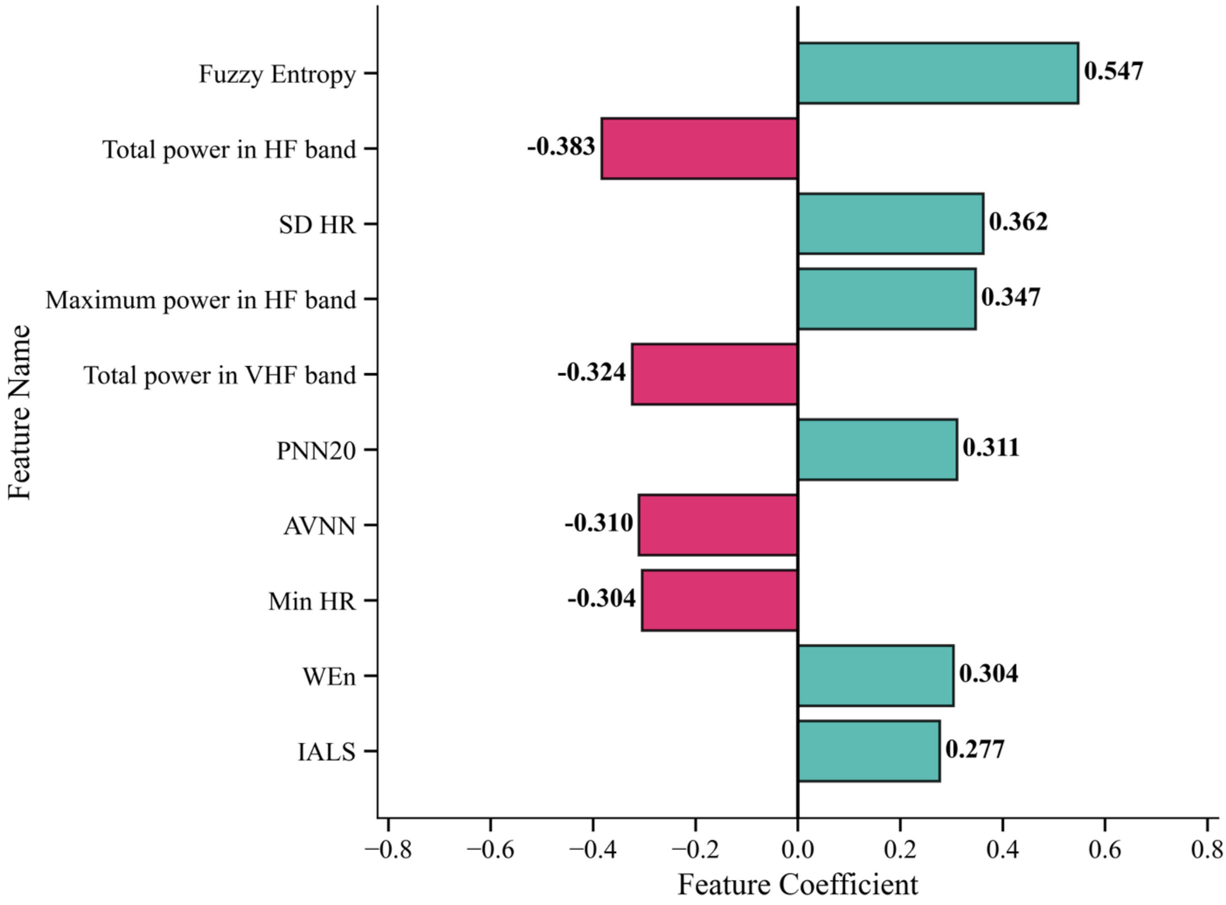

Figure S3. SHAP plot for the detection of mental stress versus seated baseline (sitting and seated recovery) for the XGBoost model. The top 10 most important features are presented in descending order of importance. Each dot represents a Shapley value for a specific instance and feature, with the color indicating the underlying feature value ranging from high (red) to low (blue). HF: high frequency (0.15–0.40 hertz); IALS: inverse of average length of the acceleration and deceleration segments; NN20: number of consecutive NN intervals differing more than 20 milliseconds (ms); PSS: the complement of the percentage of normal-to-normal (NN) intervals in acceleration or deceleration with three or more NN intervals; SHAP: Shapley additive explanation; VHF: very high frequency (0.40–0.50 hertz); XGBoost: extreme gradient boosting.

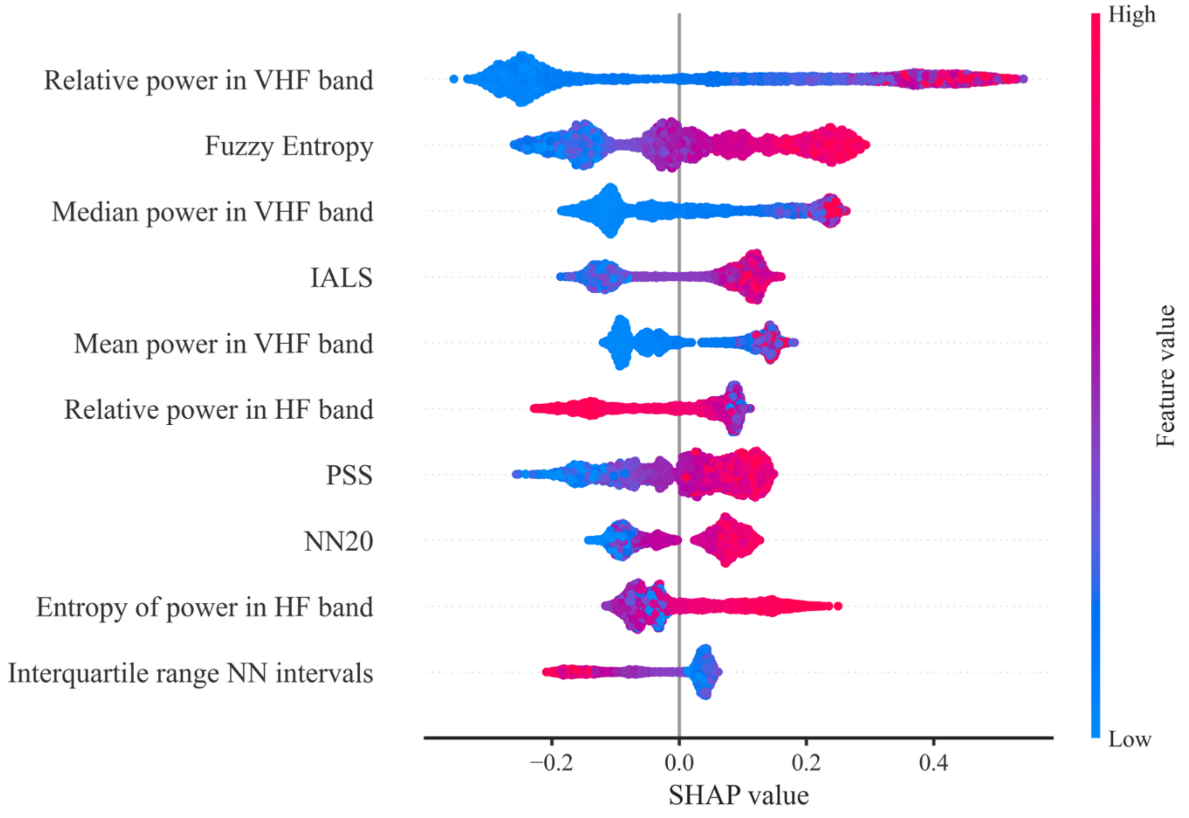

Figure S4. SHAP plot for the detection of mental stress versus seated baseline (sitting and seated recovery) for the LR model. The top 10 most important features are presented in descending order of importance. Each dot represents a Shapley value for a specific instance and feature, with the color indicating the underlying feature value ranging from high (red) to low (blue). AVNN: average value of (NN) intervals; HF: high frequency (0.15–0.40 hertz); HR: heart rate; IALS: inverse of the average length of the acceleration and deceleration segments; LR: logistic regression; PNN20: percentage consecutive normal-to-normal (NN) intervals differing by more than 20 milliseconds (ms); SD: standard deviation; SHAP: Shapley additive explanation; WEn: entropy of white vertical line length of a recurrence quantification analysis.

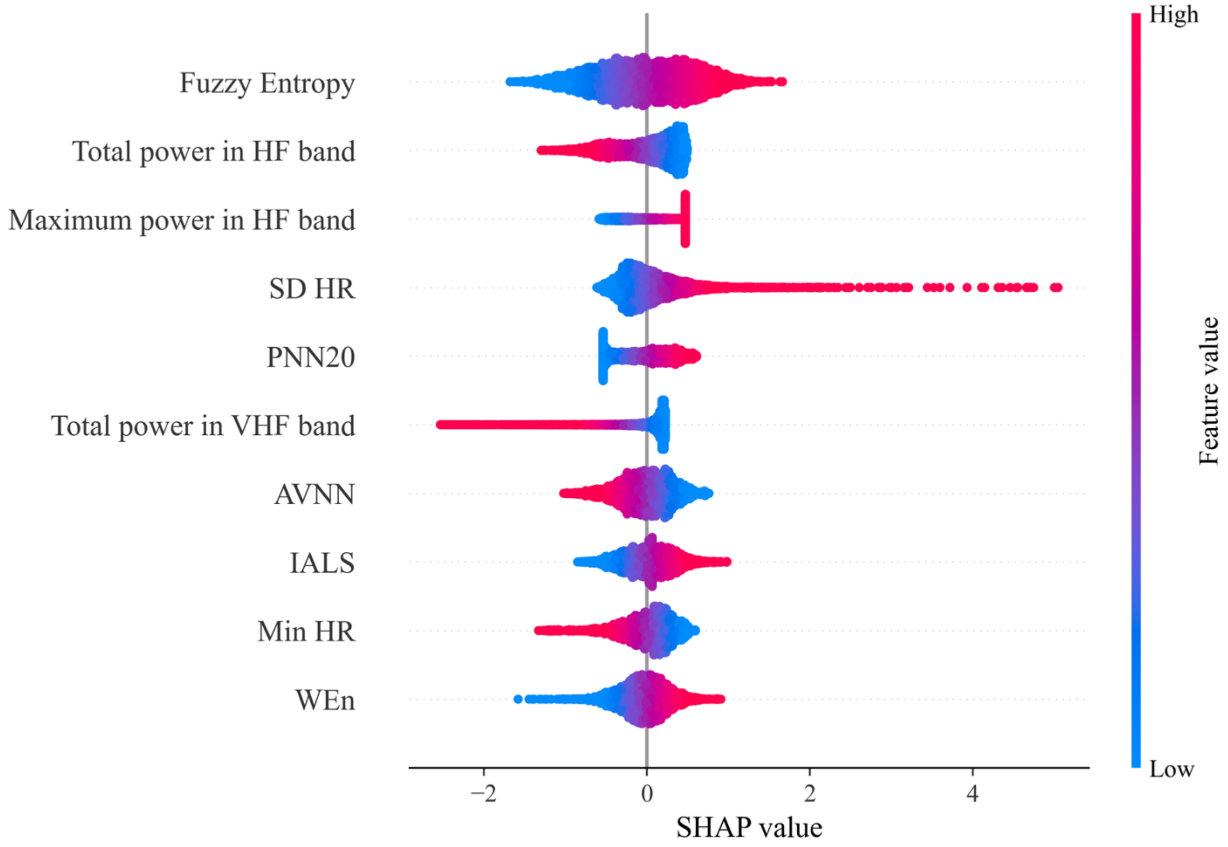

Figure S5. Top 10 features ranked by absolute coefficient magnitude in the LR model for mental stress versus seated baseline (sitting and seated recovery). AVNN: average value of (NN) intervals; HF: high frequency (0.15–0.40 hertz); HR: heart rate; LR: logistic regression; PNN20: percentage consecutive normal-to-normal (NN) intervals differing by more than 20 milliseconds (ms); PSS: The complement of the percentage of NN intervals in acceleration or deceleration with three or more NN intervals; VHF: very high frequency (0.40–0.50 hertz).

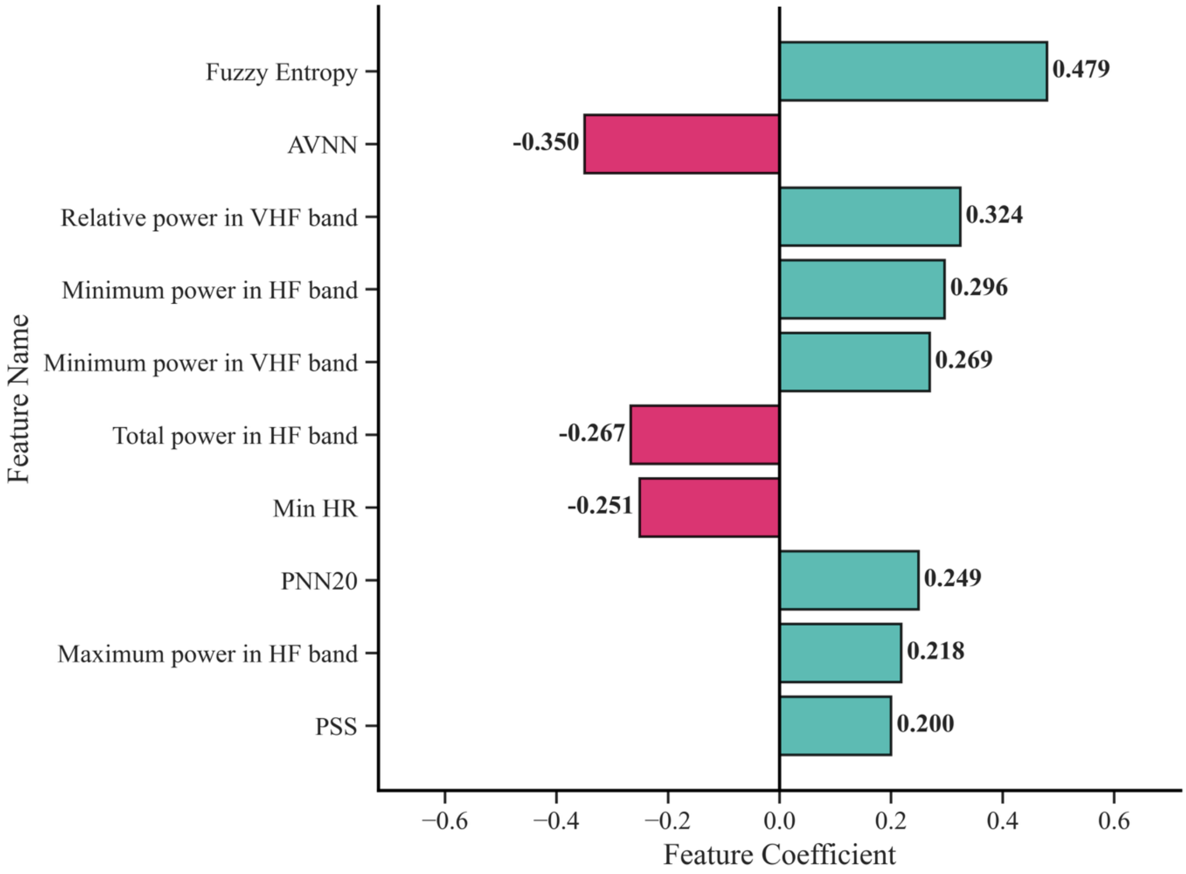

Supplement: Multimedia Appendix 9 [file jmir-v28-e80450-s009.pdf]
