# Supplementary material for: Electrocardiogram-Based Mental Stress Detection Amid Everyday Activities Using Machine Learning: Model Development and Validation Study
Source: J Med Internet Res. 2026 Apr 7;28:e80450. doi: 10.2196/80450 (PMC13055957; doi:10.2196/80450)

Feature parsimony

Figure S1. Performance comparison of LR and XGBoost for ECG-based mental stress classification across varying feature set sizes on the held-out test set (127 total participants, 26 test set participants). Points represent bootstrapped mean AUPRC with 95% CIs (error bars) based on 2000 participant-level bootstrap samples. Models were trained using a 60/20/20 (train/validation/test) split at the individual level, with features selected via forward selection based on validation performance. Both models demonstrate robustness to feature reduction, maintaining >93% of original performance with only 10 features (LR: 0.649; XGBoost: 0.688) compared to the full 55-feature set (LR: 0.691; XGBoost: 0.706). AUPRC: area under the precision-recall curve; CI: confidence interval; ECG: electrocardiogram; Hz: hertz; LR: logistic regression; XGBoost: extreme gradient boosting.

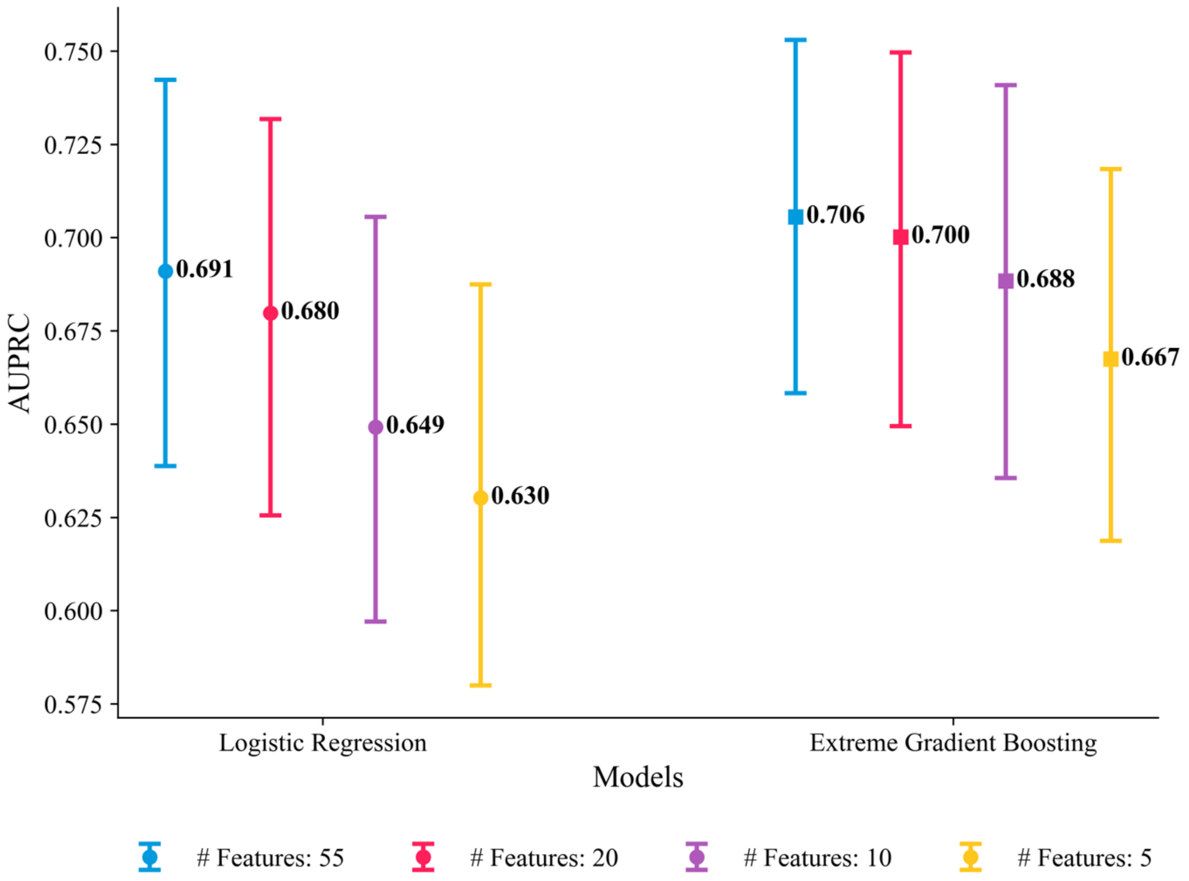

Supplement: Multimedia Appendix 10 [file jmir-v28-e80450-s010.pdf]
