# Supplementary material for: Electrocardiogram-Based Mental Stress Detection Amid Everyday Activities Using Machine Learning: Model Development and Validation Study
Source: J Med Internet Res. 2026 Apr 7;28:e80450. doi: 10.2196/80450 (PMC13055957; doi:10.2196/80450)
Supplement: Multimedia Appendix 11 [file jmir-v28-e80450-s011.pdf]

Overview of selected features

In this section, we provide additional information on the selected features chosen during the feature selection process for both the logistic regression (LR) and extreme gradient boosting (XGBoost) models. As stated in the main text, models were trained on data from 127 participants using a 60/20/20 (train/validation/test) split at the subject level.

We evaluated feature sets of 20, 10, and 5 features. Features were selected via forward selection, iteratively adding features based on their contribution to validation set performance. This process was performed independently for each model to identify the most informative features for mental stress detection.

Logistic Regression

Top 20 selected features:
Min HR, Mean HR, SD HR, AVNN, SDNN, IQR NN, NN20, PNN20, Min power in HF band, Entropy in HF band, Total band power in HF band, Min power in VHF band, Relative band power in VHF band, Min power in UHF band, Max power in UHF band, DFA Alpha1, Fuzzy Entropy, W, Wen, PSS.

Top 10 selected features:
Min HR, SD HR, AVNN, SDNN, NN20, Min power in HF band, Total power in HF band, Min power in UHF band, Fuzzy entropy, PSS.

Top 5 selected features:
Min HR, AVNN, NN20, Fuzzy entropy, PSS.

Extreme Gradient Boosting

Top 20 selected features:
Min HR, Max HR, RMSSD, NN20, PNN20, Min power in HF band, relative band power in HF band, Min power in VHF band, Max power in VHF band, Median power in VHF band, Mean power in VHF band, Relative band power in VHF band, Min power in VHF band, Min power in UHF band, Fuzzy entropy, SD1, Wen, PSS, PIP, IALS.

Top 10 selected features:
NN20, PNN20, Min power in VHF band, Median power in VHF band, Mean power in VHF band, Total band power in VHF band, Relative band power in VHF band, Fuzzy entropy, PSS, IALS.

Top 5 selected features:
PNN20, Median power in VHF band, Mean power in VHF band, Total band power VHF band, IALS.
