# Supplementary material for: Electrocardiogram-Based Mental Stress Detection Amid Everyday Activities Using Machine Learning: Model Development and Validation Study
Source: J Med Internet Res. 2026 Apr 7;28:e80450. doi: 10.2196/80450 (PMC13055957; doi:10.2196/80450)
Supplement: Multimedia Appendix 12 [file jmir-v28-e80450-s012.pdf]

Generalization to unknown stressors

Table S1. Bootstrapped mean AUPRC with 95% CIs from 2000 participant-level bootstrap samples for binary classification of mental stress versus non-stress conditions under leave-one-stressor-out evaluation on the held-out test set (127 total participants, 26 test set participants). Models were trained using a 60/20/20 (train/validation/test) split at the participant level, using a total of 55 features. For each row, the "Baseline" column reports the model's AUPRC on stressors included in the training set. In contrast, left-out reports the AUPRC model performance on the held-out (unseen) mental stressor, assessing each model's ability to generalize to novel mental stress conditions. The bootstrapped AUPRC performance of a random classifier that predicts the majority class is 0.46 [0.40, 0.52] for the SSST and 0.46 [0.43, 0.48] for the other mental stressors. Note that if a mental stressor had a repeat condition in the experimental protocol (eg., TA, PASAT), both stressors were left out during training of the machine learning models, which is why their baseline scores are identical. AUPRC: area under the precision-recall curve; CI: confidence interval; PASAT: paced auditory serial addition task; SSST: sing-a-song-stress test; TA: tone avoidance.

Left-out mental stressor	AUPRC Model Performance, 95% CI					
	**LR[a] baseline**	**LR left-out**	**XGBoost[b] baseline**	**XGBoost left-out**	**RF[c] baseline**	**RF left-out**
SSST[d]	0.6885 (0.6363–0.7396)	0.5122 (0.4081–0.6235)	0.6971 (0.6505–0.7451)	0.5404 (0.4346–0.6583)	0.7099 (0.6629–0.7571)	0.5282 (0.4229–0.6490)
Raven[e]	0.6710 (0.6225–0.7179)	0.5537 (0.4896–0.6314)	0.6896 (0.6504–0.7280)	0.6004 (0.5452–0.6593)	0.7013 (0.6633–0.7380)	0.6196 (0.5616–0.6766)
Pasat[f]	0.6962 (0.6460–0.7444)	0.6631 (0.5907–0.7339)	0.7036 (0.6567–0.7496)	0.6836 (0.6209–0.7487)	0.7096 (0.6617–0.7557)	0.6910 (0.6248–0.7575)
Pasat (repeat)	0.6962 (0.6460–0.7444)	0.6581 (0.5760–0.7361)	0.7036 (0.6567–0.7496)	0.6596 (0.5838–0.7302)	0.7096 (0.6617–0.7557)	0.6762 (0.6016–0.7474)
TA[g]	0.6806 (0.6328–0.7313)	0.7427 (0.6778–0.8071)	0.6706 (0.6272–0.7144)	0.7015 (0.6388–0.7677)	0.6766 (0.6312–0.7230)	0.7163 (0.6503–0.7814)
TA (repeat)	0.6806 (0.6328–0.7313)	0.7315 (0.6567–0.8042)	0.6706 (0.6272–0.7144)	0.7085 (0.6398–0.7746)	0.6766 (0.6312–0.7230)	0.7247 (0.6476–0.7940)

[a]LR: logistic regression.
[b]XGBoost: extreme gradient boosting.
[c]RF: random forest.
[d]SSST: sing-a-song-stress test.
[e]RAVEN: Raven's progressive matrices.
[f]PASAT: paced auditory serial addition task.
[g]TA: tone avoidance.
