# Supplementary material for: Electrocardiogram-Based Mental Stress Detection Amid Everyday Activities Using Machine Learning: Model Development and Validation Study
Source: J Med Internet Res. 2026 Apr 7;28:e80450. doi: 10.2196/80450 (PMC13055957; doi:10.2196/80450)
Supplement: Multimedia Appendix 13 [file jmir-v28-e80450-s013.pdf]

Stratified stressor analysis

Table S1. Bootstrapped mean AUPRC with 95% CIs from 2000 participant-level bootstrap samples for mental stress classification, stratified by individual stressor type and evaluated on the held-out test set (127 total participants, 26 test set participants). Results shown for LR, XGBoost, and RF, trained using a 60/20/20 (train/validation/test) split at the individual level with 55 features. Each row presents model performance for a specific mental stressor, assessing how well the models detect that stressor. We further report the performance of a random baseline that predicts the majority class. AUPRC: area under the precision-recall curve; CI: confidence interval; LR: logistic regression; RF: random forest; XGBoost: extreme gradient boosting.

**Mental stressor**	**AUPRC Model Performance, 95% CI**			
	**LR**[a]	**XGBoost**[b]	**RF**[c]	**Random BL**[d]
SSST[e]	0.5290 (0.4286–0.6360)	0.5442 (0.4371–0.6632)	0.5332 (0.4309–6498)	0.4584 (0.3980–5230)
RAVEN[f]	0.5777 (0.5140–0.6528)	0.6282 (0.5682–0.6857)	0.6299 (0.5722–0.6882)	0.4583 (0.4402–0.4782)
PASAT[g]	0.6622 (0.5888–0.7364)	0.6930 (0.6287–0.7595)	0.6854 (0.6083–0.7586)	0.4583 (0.4402–0.4783)
PASAT (repeat)	0.6612 (0.5778–0.7398)	0.6685 (0.5957–0.7385)	0.6921 (0.6271–0.7587)	0.4583 (0.4402–0.4783)
TA[h]	0.7663 (0.7050–0.8274)	0.7756 (0.7241–0.8251)	0.7832 (0.7276–0.8362)	0.4582 (0.4399–0.4783)
TA (repeat)	0.7618 (0.6884–0.8302)	0.7867 (0.7208–0.8472)	0.7923 (0.7246–0.8561)	0.4580 (0.4396–0.4780)

[a]LR: logistic regression.
[b]XGBoost: extreme gradient boosting.
[c]RF: random forest.
[d]BL: baseline.
[e]SSST: sing-a-song-stress test.
[f]RAVEN: Raven's progressive matrices.
[g]PASAT: paced auditory serial addition task.
[h]TA: tone avoidance.
