# Supplementary material for: Electrocardiogram-Based Mental Stress Detection Amid Everyday Activities Using Machine Learning: Model Development and Validation Study
Source: J Med Internet Res. 2026 Apr 7;28:e80450. doi: 10.2196/80450 (PMC13055957; doi:10.2196/80450)
Supplement: Multimedia Appendix 14 [file jmir-v28-e80450-s014.pdf]

Stratified Stressor Analysis: Single Stressor Training

Table S1. Bootstrapped mean AUROC with 95% CIs from 2000 participant-level bootstrap samples for stressor-specific classification against the non-stressed condition. Results on the held-out test set (127 total participants, 26 test set participants) are shown for LR and XGBoost. Each model was independently trained to distinguish one mental stressor from the baseline using a 60/20/20 (train/validation/test) split at the individual level, with 55 features. The performance of a random classifier predicting the majority class is 0.50. AUROC: area under the receiver operating characteristic; CI: confidence interval; LR: logistic regression; XGBoost: extreme gradient boosting.

**Mental stressor**	**AUROC Model Performance, 95% CI**	
	**LR**[a]	**XGBoost**[b]
SSST[c]	0.7341 (0.6607–0.7983)	0.7611 (0.6967–0.8187)
RAVEN[d]	0.7371 (0.6967–0.7763)	0.7414 (0.7005–0.7827)
PASAT[e]	0.7131 (0.6453–0.7777)	0.7121 (0.6510–0.7724)
PASAT (repeat)	0.7094 (0.6381–0.7763)	0.7256 (0.6557–0.7856)
TA[f]	0.8163 (0.7741–0.8604)	0.8306 (0.7930–0.8668)
TA (repeat)	0.8394 (0.7794–0.8887)	0.8449 (0.7814–0.8976)

[a]LR: logistic regression.
[b]XGBoost: extreme gradient boosting.
[c]SSST: sing-a-song-stress test.
[d]RAVEN: Raven's progressive matrices.
[e]PASAT: paced auditory serial addition task.
[f]TA: tone avoidance.

Stratified Stressor Analysis—Single Stressor Training

Table S2. Bootstrapped mean AUPRC with 95% CIs from 2000 participant-level bootstrap samples for stressor-specific classification against the non-stressed condition. Results on the held-out test set (127 total participants, 26 test set participants) are shown for LR and XGBoost. Each model was independently trained to distinguish one mental stressor from the baseline using a 60/20/20 (train/validation/test) split at the individual level, with 55 features. We further report the performance of a random baseline that predicts the majority class. AUPRC: area under the precision-recall curve; CI: confidence interval; LR: logistic regression; XGBoost: extreme gradient boosting.

Mental stressor	AUPRC Model Performance, 95% CI		
	LR[a]	XGBoost[b]	Random BL[c]
SSST[d]	0.0812 (0.0461–0.1506)	0.0948 (0.0549–0.1516)	0.0229 (0.0208–0.0247)
RAVEN[e]	0.2785 (0.2290–0.3399)	0.2888 (0.2382–0.3460)	0.1405 (0.1391–0.1423)
PASAT[f]	0.3174 (0.2530–0.3919)	0.3043 (0.2495–0.3629)	0.1405 (0.1384–0.1426)
PASAT (repeat)	0.3299 (0.2399–0.4313)	0.3104 (0.2210–0.4110)	0.1401 (0.1373–0.1426)
TA[g]	0.4715 (0.3806–0.5753)	0.4658 (0.3994–0.5390)	0.1424 (0.1399–0.1450)
TA (repeat)	0.4973 (0.4018–0.5928)	0.5060 (0.3988–0.6054)	0.1422 (0.1402–0.1442)

[a]LR: logistic regression.
[b]XGBoost: extreme gradient boosting.
[c]BL: baseline.
[d]SSST: sing-a-song-stress test.
[e]RAVEN: Raven’s progressive matrices.
[f]PASAT: paced auditory serial addition task.
[g]TA: tone avoidance.
